# Supplementary material for: Apelin-VEGF-C mRNA delivery as therapeutic for the treatment of secondary lymphedema
Source: EMBO Mol Med. 2024 Jan 2;16(2):386–415. doi: 10.1038/s44321-023-00017-7 (PMC10898257; doi:10.1038/s44321-023-00017-7)
Supplement: Supplementary file 2 — Movie EV1 [file 44321_2023_17_MOESM2_ESM.zip › Movie EV1.docx]

**Movie EV1. Limb collecting vessel contraction in WT mice.**

Vessel contraction measurement of afferent collecting lymphatic vessels to the popliteal lymph node
