# Supplementary figures and images for: Apelin-VEGF-C mRNA delivery as therapeutic for the treatment of secondary lymphedema
Source: EMBO Mol Med. 2024 Jan 2;16(2):386–415. doi: 10.1038/s44321-023-00017-7 (PMC10898257; doi:10.1038/s44321-023-00017-7)

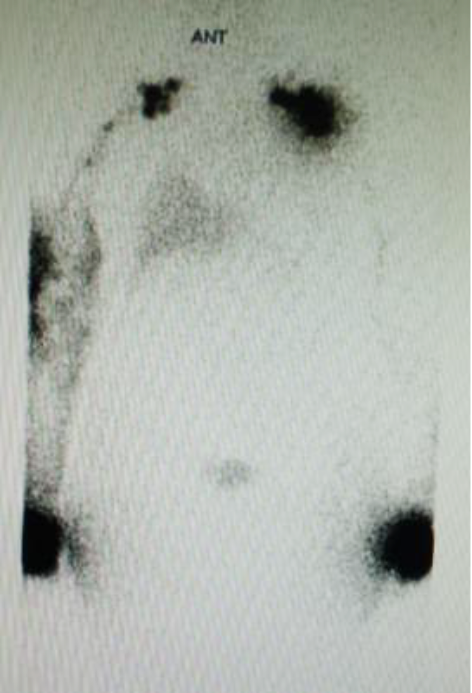

Supplement: Supplementary file 5 — Source Data Fig. 1 [file 44321_2023_17_MOESM5_ESM.zip › Figure 1/Panel 1C.tif]

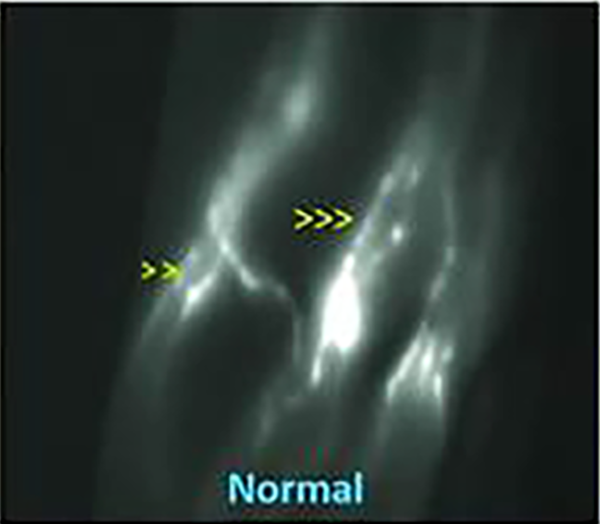

Supplement: Supplementary file 5 — Source Data Fig. 1 [file 44321_2023_17_MOESM5_ESM.zip › Figure 1/Panel1A left.tiff]

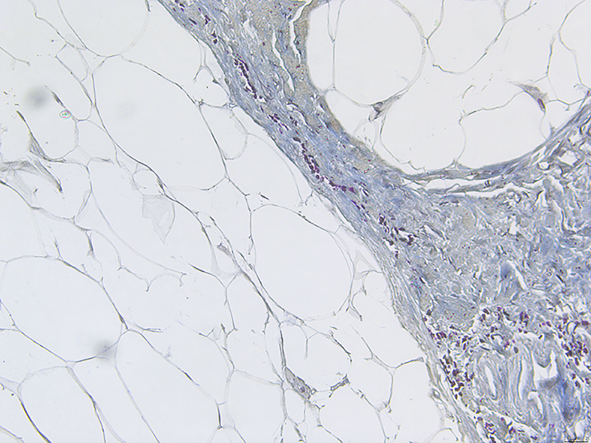

Supplement: Supplementary file 5 — Source Data Fig. 1 [file 44321_2023_17_MOESM5_ESM.zip › Figure 1/1G.tif]

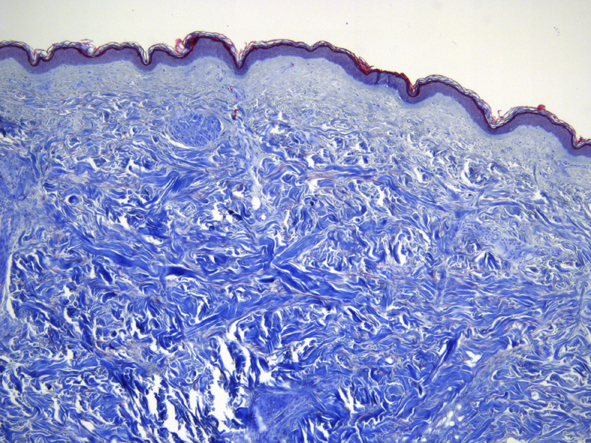

Supplement: Supplementary file 5 — Source Data Fig. 1 [file 44321_2023_17_MOESM5_ESM.zip › Figure 1/1F right.tif]

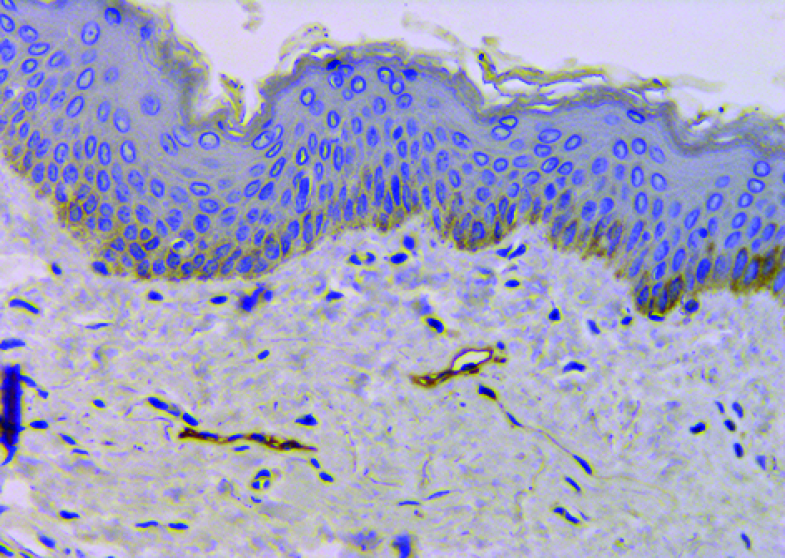

Supplement: Supplementary file 5 — Source Data Fig. 1 [file 44321_2023_17_MOESM5_ESM.zip › Figure 1/1C left.tif]

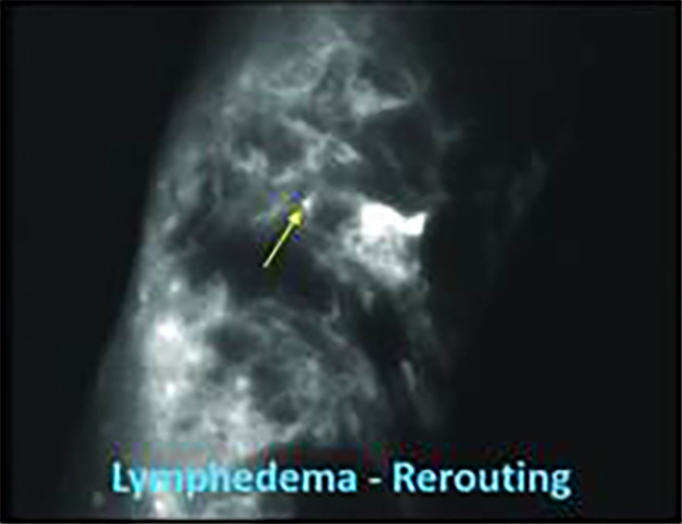

Supplement: Supplementary file 5 — Source Data Fig. 1 [file 44321_2023_17_MOESM5_ESM.zip › Figure 1/Panel1B right.tif]

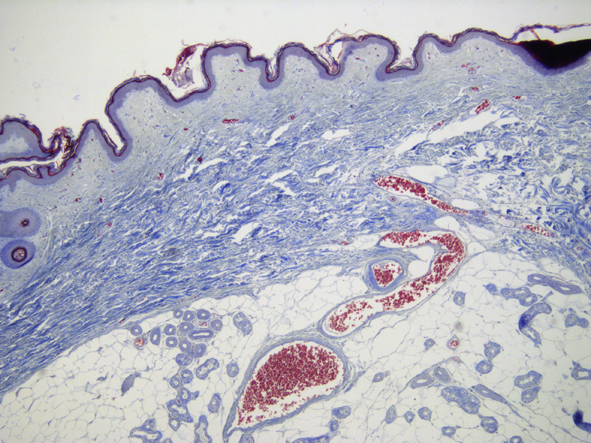

Supplement: Supplementary file 5 — Source Data Fig. 1 [file 44321_2023_17_MOESM5_ESM.zip › Figure 1/1F left.tif]

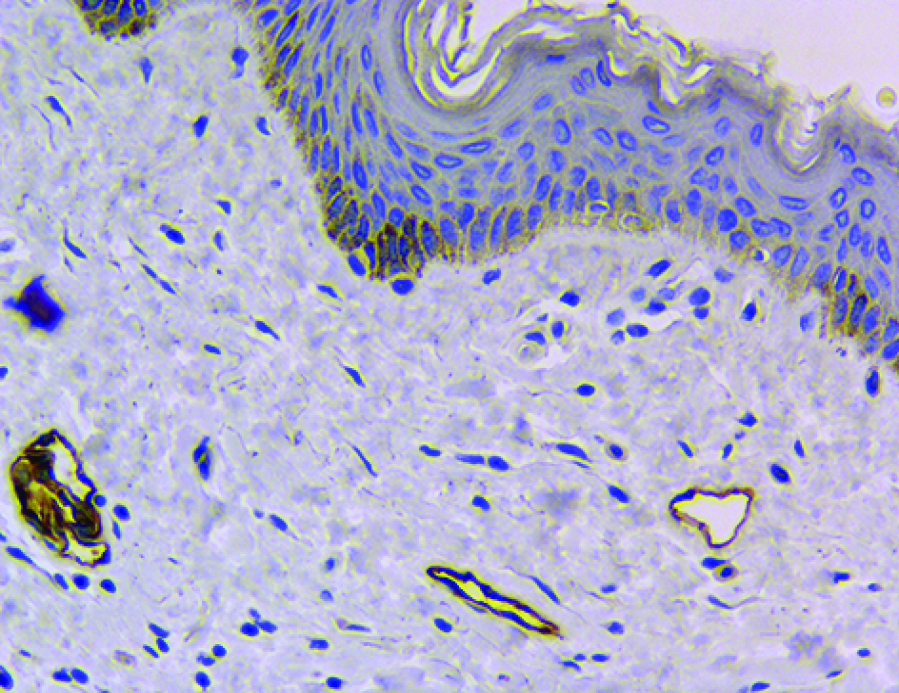

Supplement: Supplementary file 5 — Source Data Fig. 1 [file 44321_2023_17_MOESM5_ESM.zip › Figure 1/1C right.tif]

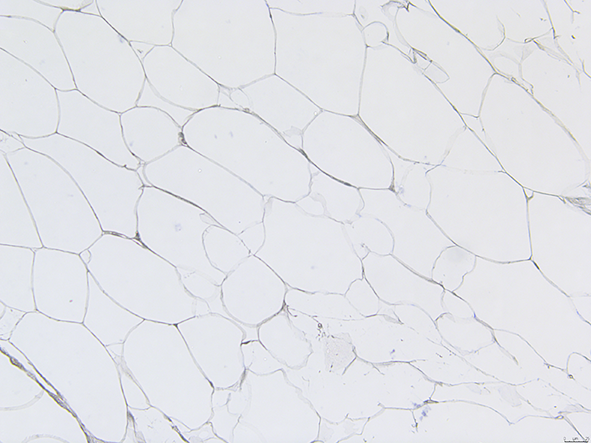

Supplement: Supplementary file 5 — Source Data Fig. 1 [file 44321_2023_17_MOESM5_ESM.zip › Figure 1/1Gleft.tif]

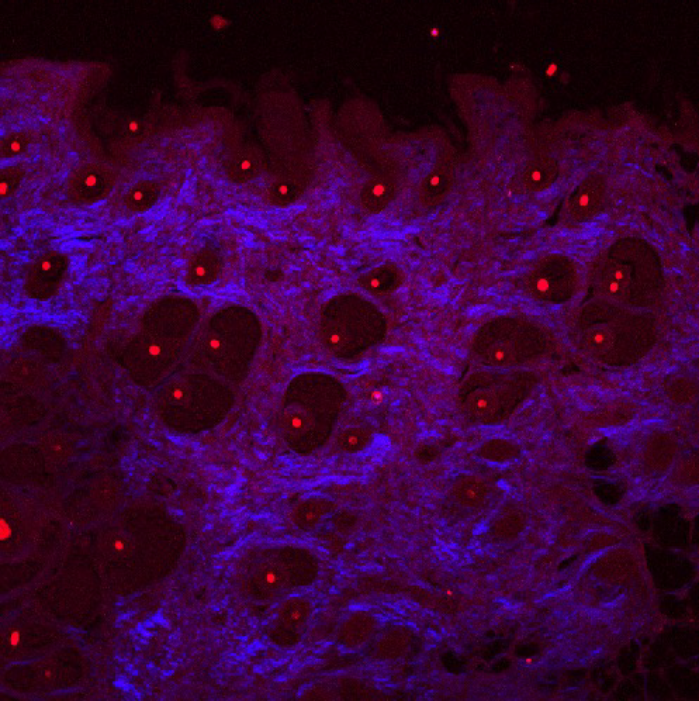

Supplement: Supplementary file 6 — Source Data Fig. 2 [file 44321_2023_17_MOESM6_ESM.zip › Figure 2/2G4.tif]

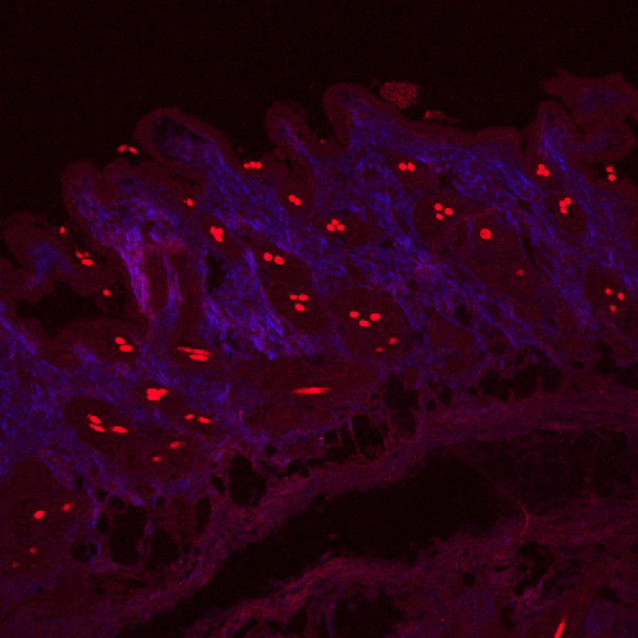

Supplement: Supplementary file 6 — Source Data Fig. 2 [file 44321_2023_17_MOESM6_ESM.zip › Figure 2/2G2.tif]

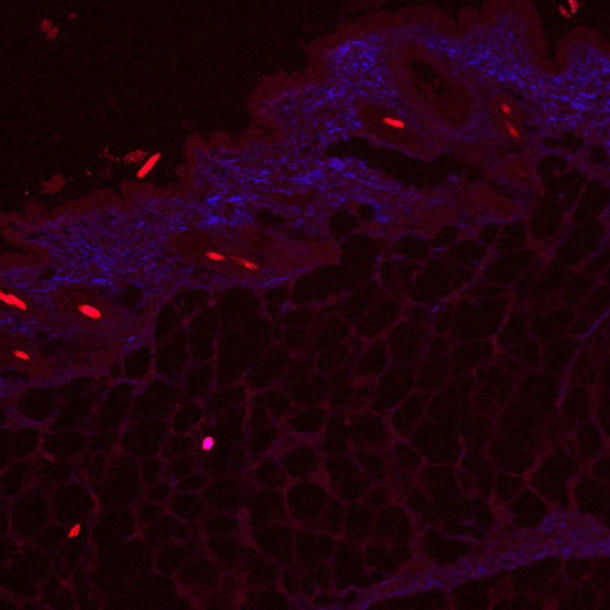

Supplement: Supplementary file 6 — Source Data Fig. 2 [file 44321_2023_17_MOESM6_ESM.zip › Figure 2/2G3.tif]

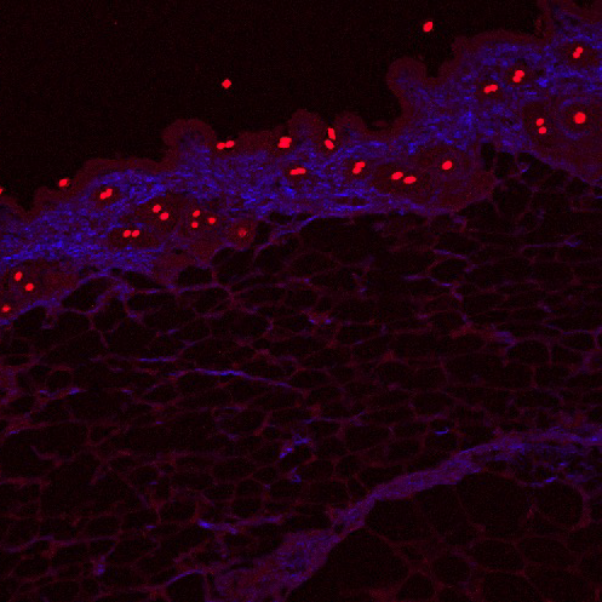

Supplement: Supplementary file 6 — Source Data Fig. 2 [file 44321_2023_17_MOESM6_ESM.zip › Figure 2/2G1.tif]

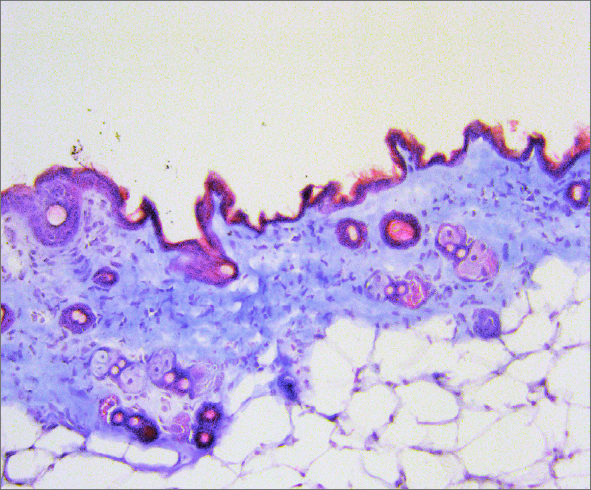

Supplement: Supplementary file 6 — Source Data Fig. 2 [file 44321_2023_17_MOESM6_ESM.zip › Figure 2/2E3.tif]

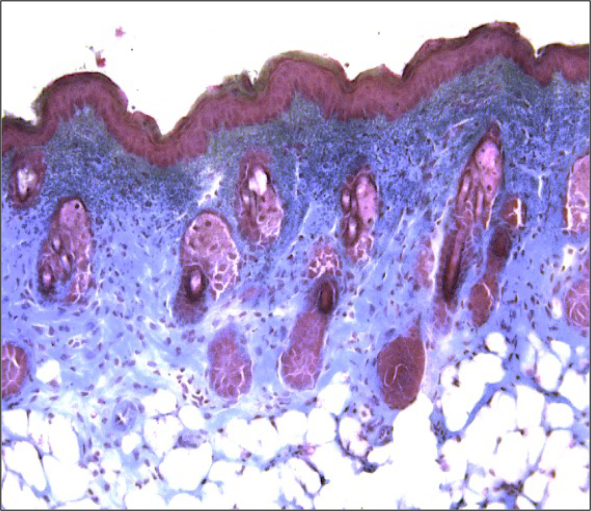

Supplement: Supplementary file 6 — Source Data Fig. 2 [file 44321_2023_17_MOESM6_ESM.zip › Figure 2/2E2.tif]

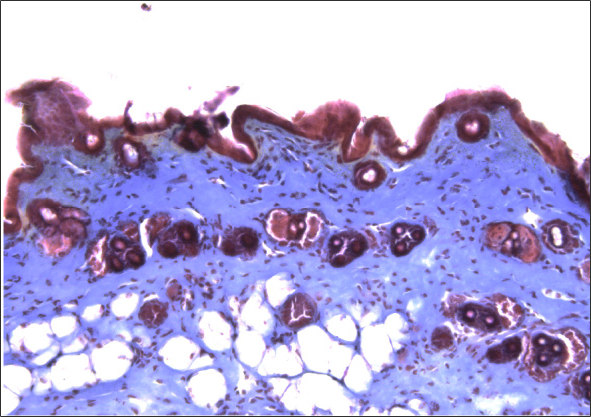

Supplement: Supplementary file 6 — Source Data Fig. 2 [file 44321_2023_17_MOESM6_ESM.zip › Figure 2/2E1.tif]

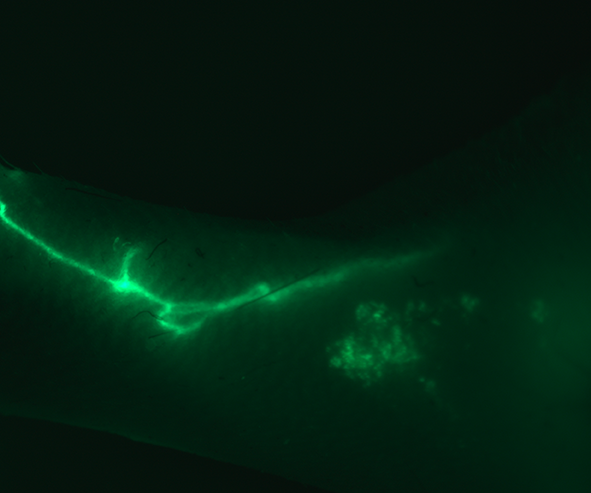

Supplement: Supplementary file 6 — Source Data Fig. 2 [file 44321_2023_17_MOESM6_ESM.zip › Figure 2/2B2.tif]

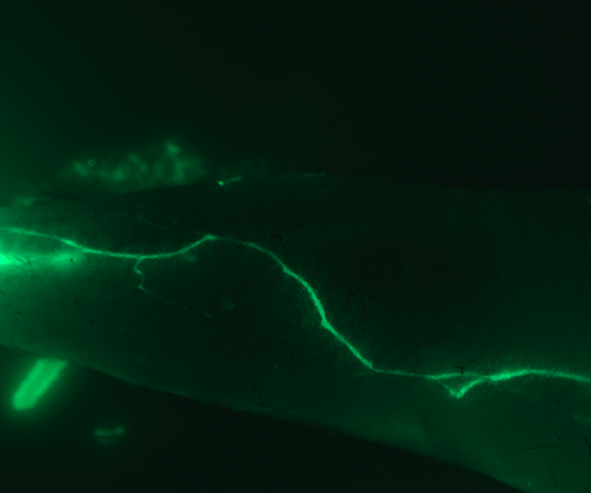

Supplement: Supplementary file 6 — Source Data Fig. 2 [file 44321_2023_17_MOESM6_ESM.zip › Figure 2/2B3.tif]

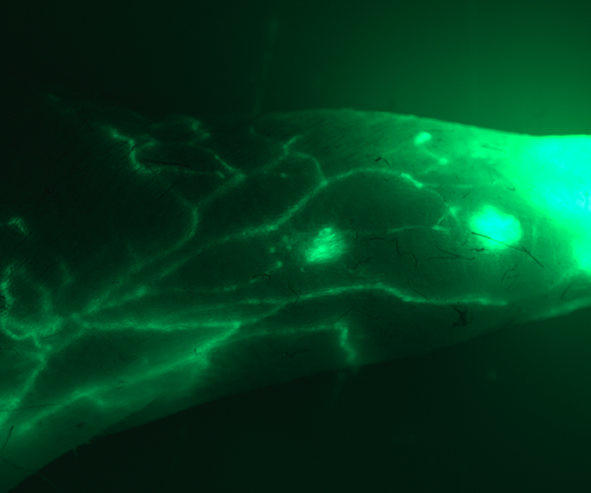

Supplement: Supplementary file 6 — Source Data Fig. 2 [file 44321_2023_17_MOESM6_ESM.zip › Figure 2/2B1.tif]

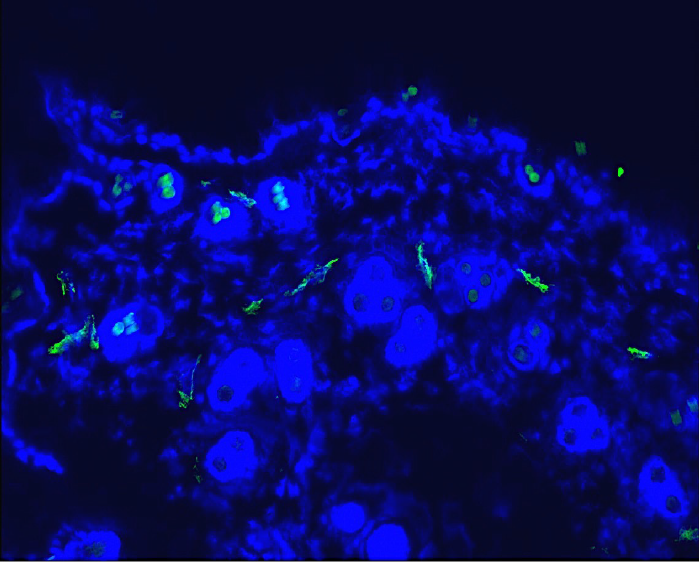

Supplement: Supplementary file 6 — Source Data Fig. 2 [file 44321_2023_17_MOESM6_ESM.zip › Figure 2/2C4.tif]

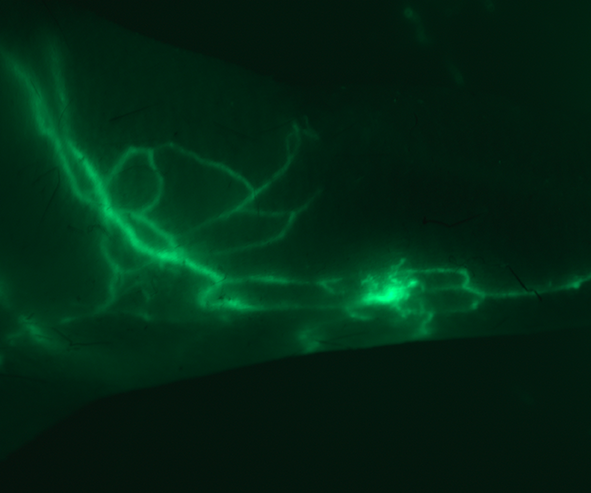

Supplement: Supplementary file 6 — Source Data Fig. 2 [file 44321_2023_17_MOESM6_ESM.zip › Figure 2/2B4.tif]

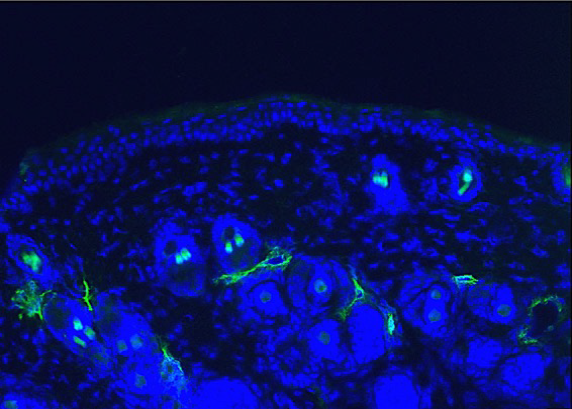

Supplement: Supplementary file 6 — Source Data Fig. 2 [file 44321_2023_17_MOESM6_ESM.zip › Figure 2/2C1.tif]

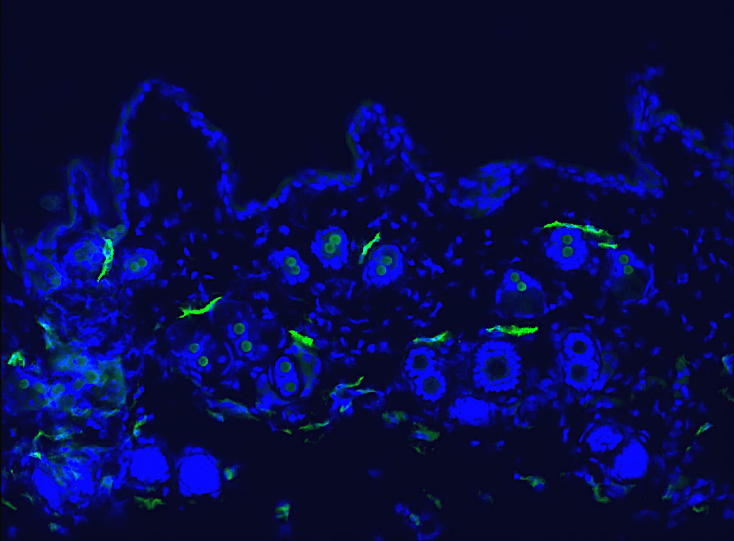

Supplement: Supplementary file 6 — Source Data Fig. 2 [file 44321_2023_17_MOESM6_ESM.zip › Figure 2/2C3.tif]

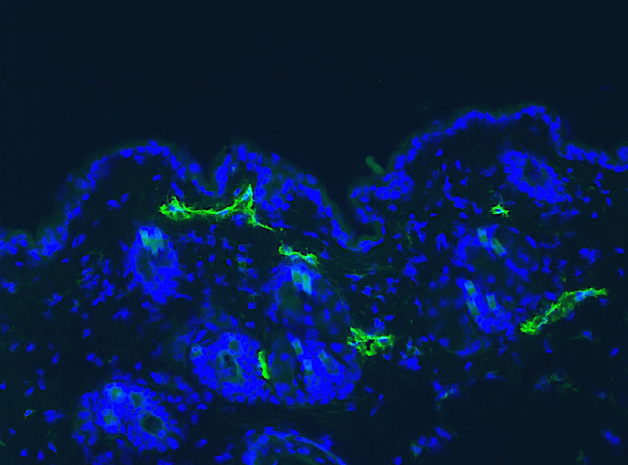

Supplement: Supplementary file 6 — Source Data Fig. 2 [file 44321_2023_17_MOESM6_ESM.zip › Figure 2/2C2.tif]

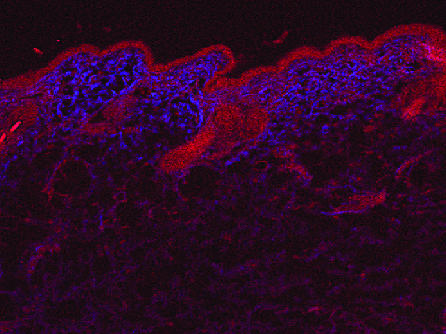

Supplement: Supplementary file 7 — Source Data Fig. 3 [file 44321_2023_17_MOESM7_ESM.zip › Figure 3/3G4.tif]

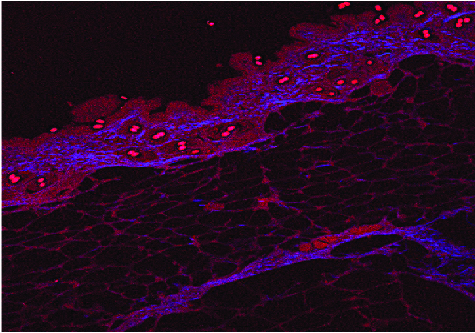

Supplement: Supplementary file 7 — Source Data Fig. 3 [file 44321_2023_17_MOESM7_ESM.zip › Figure 3/3G1.tif]

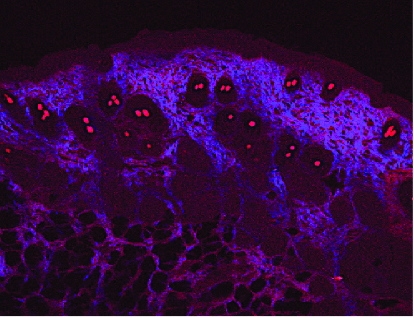

Supplement: Supplementary file 7 — Source Data Fig. 3 [file 44321_2023_17_MOESM7_ESM.zip › Figure 3/3G2.tif]

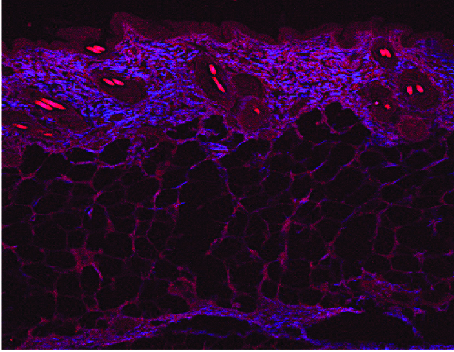

Supplement: Supplementary file 7 — Source Data Fig. 3 [file 44321_2023_17_MOESM7_ESM.zip › Figure 3/3G3.tif]

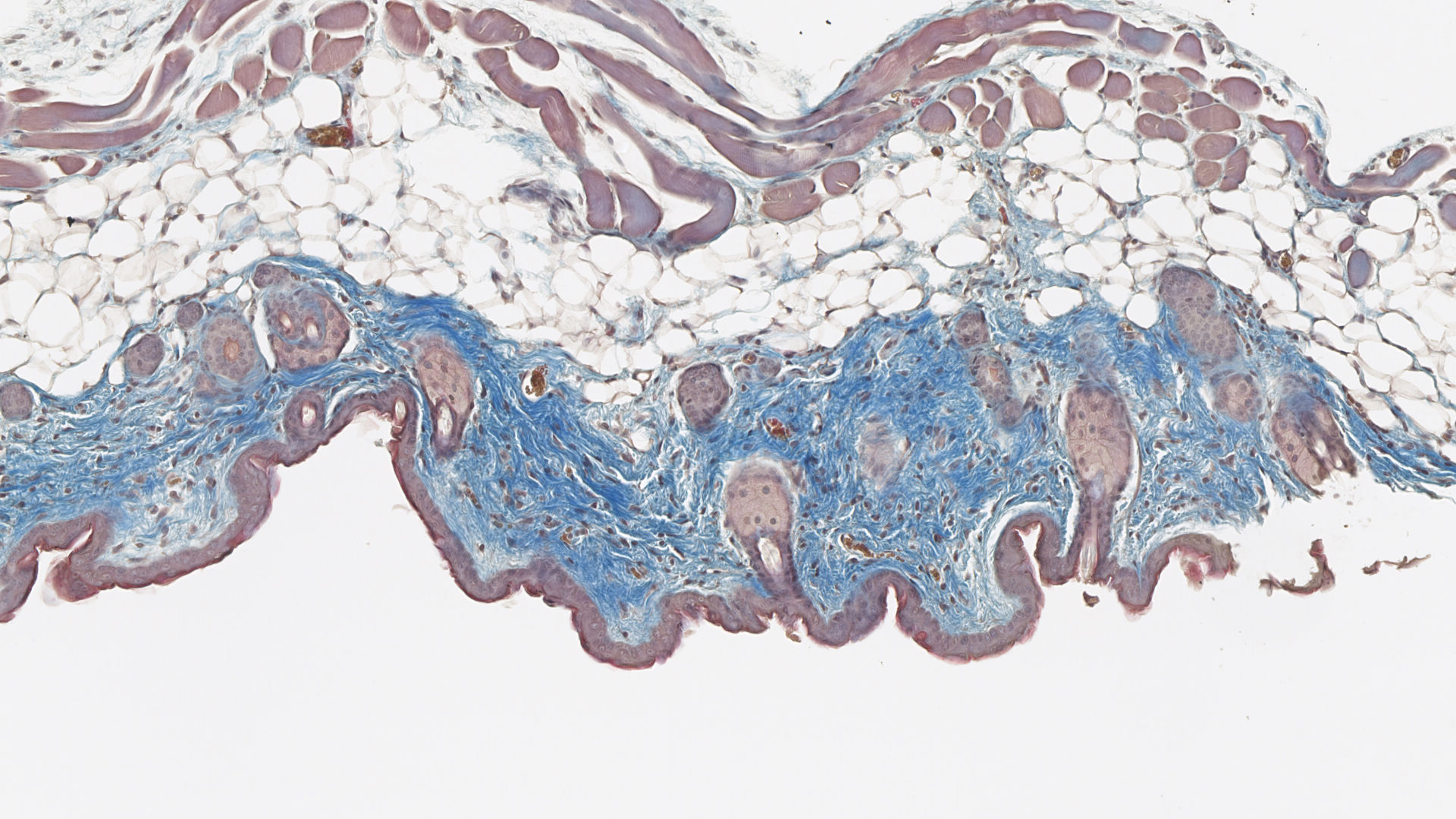

Supplement: Supplementary file 7 — Source Data Fig. 3 [file 44321_2023_17_MOESM7_ESM.zip › Figure 3/3D4.jpg]

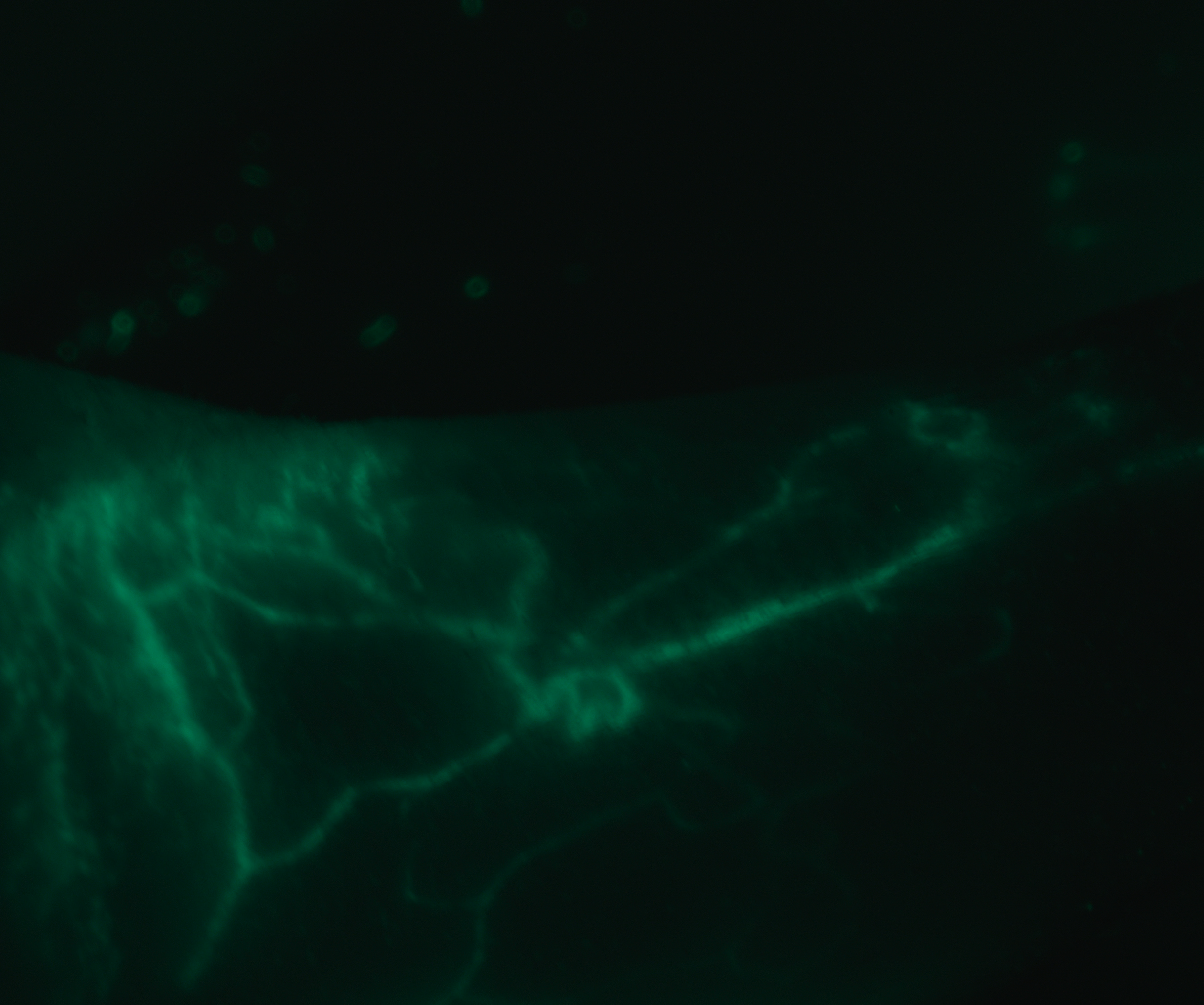

Supplement: Supplementary file 7 — Source Data Fig. 3 [file 44321_2023_17_MOESM7_ESM.zip › Figure 3/3C4.tif]

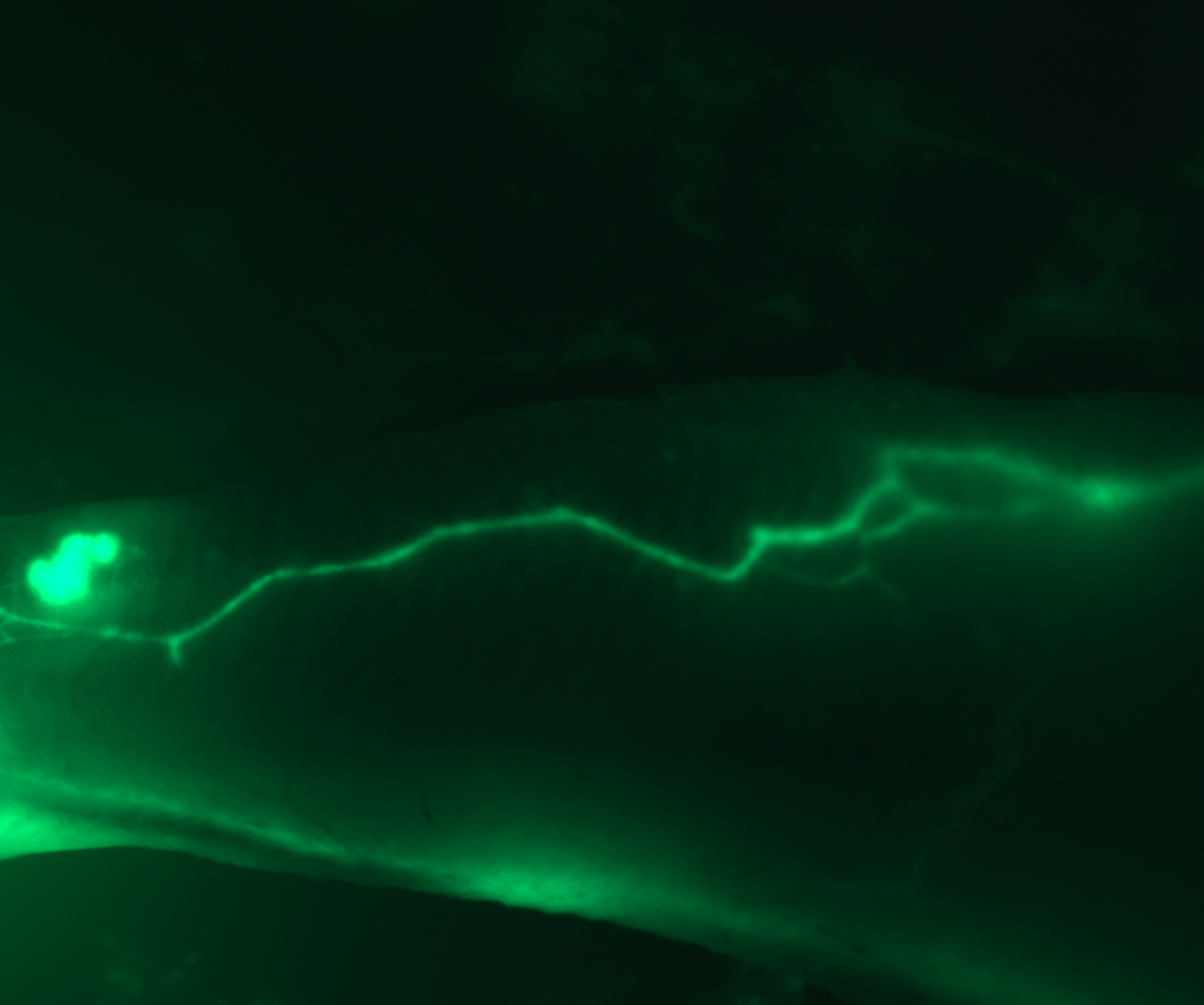

Supplement: Supplementary file 7 — Source Data Fig. 3 [file 44321_2023_17_MOESM7_ESM.zip › Figure 3/3C3.tif]

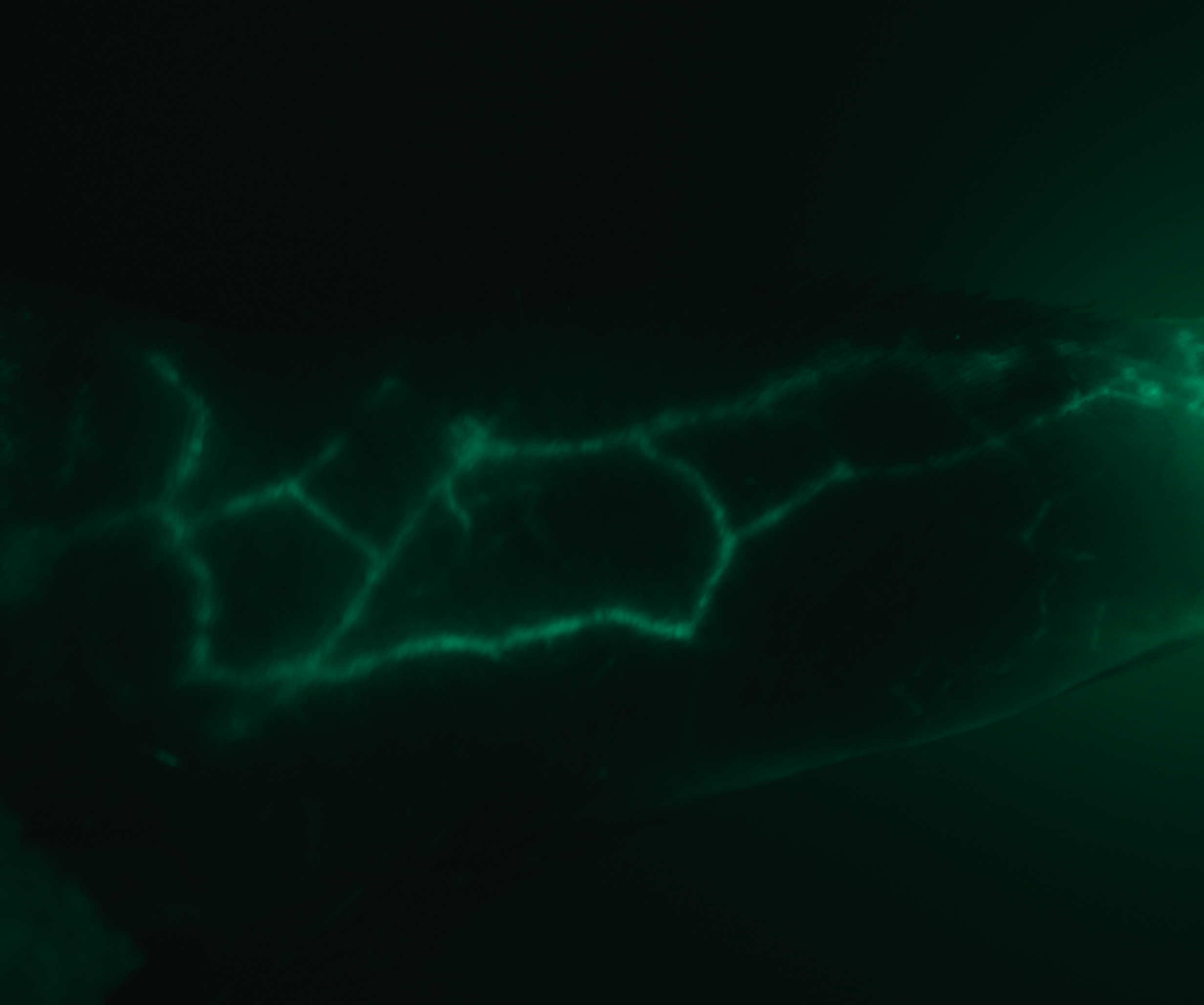

Supplement: Supplementary file 7 — Source Data Fig. 3 [file 44321_2023_17_MOESM7_ESM.zip › Figure 3/3C2.tif]

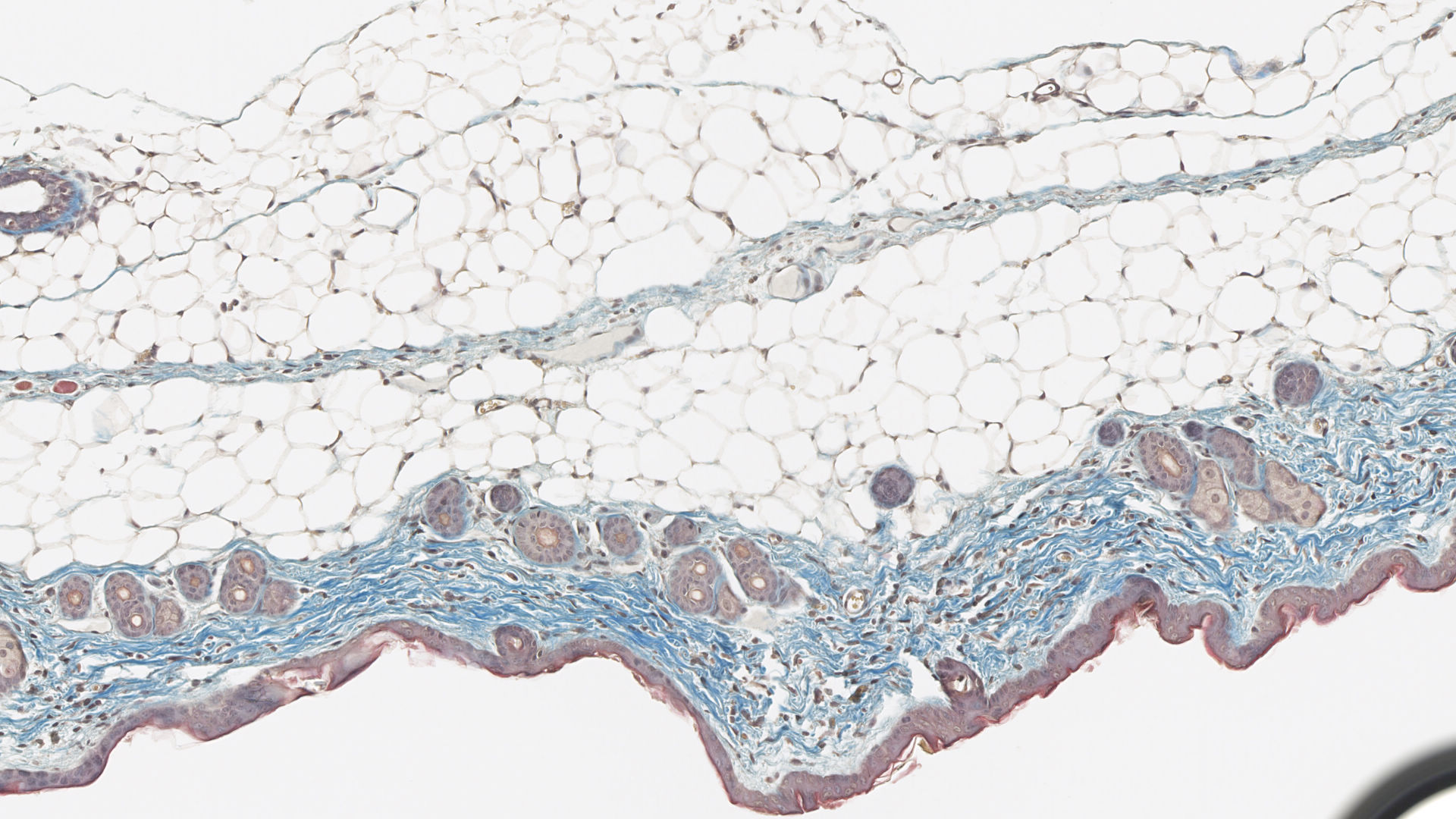

Supplement: Supplementary file 7 — Source Data Fig. 3 [file 44321_2023_17_MOESM7_ESM.zip › Figure 3/3D1.jpg]

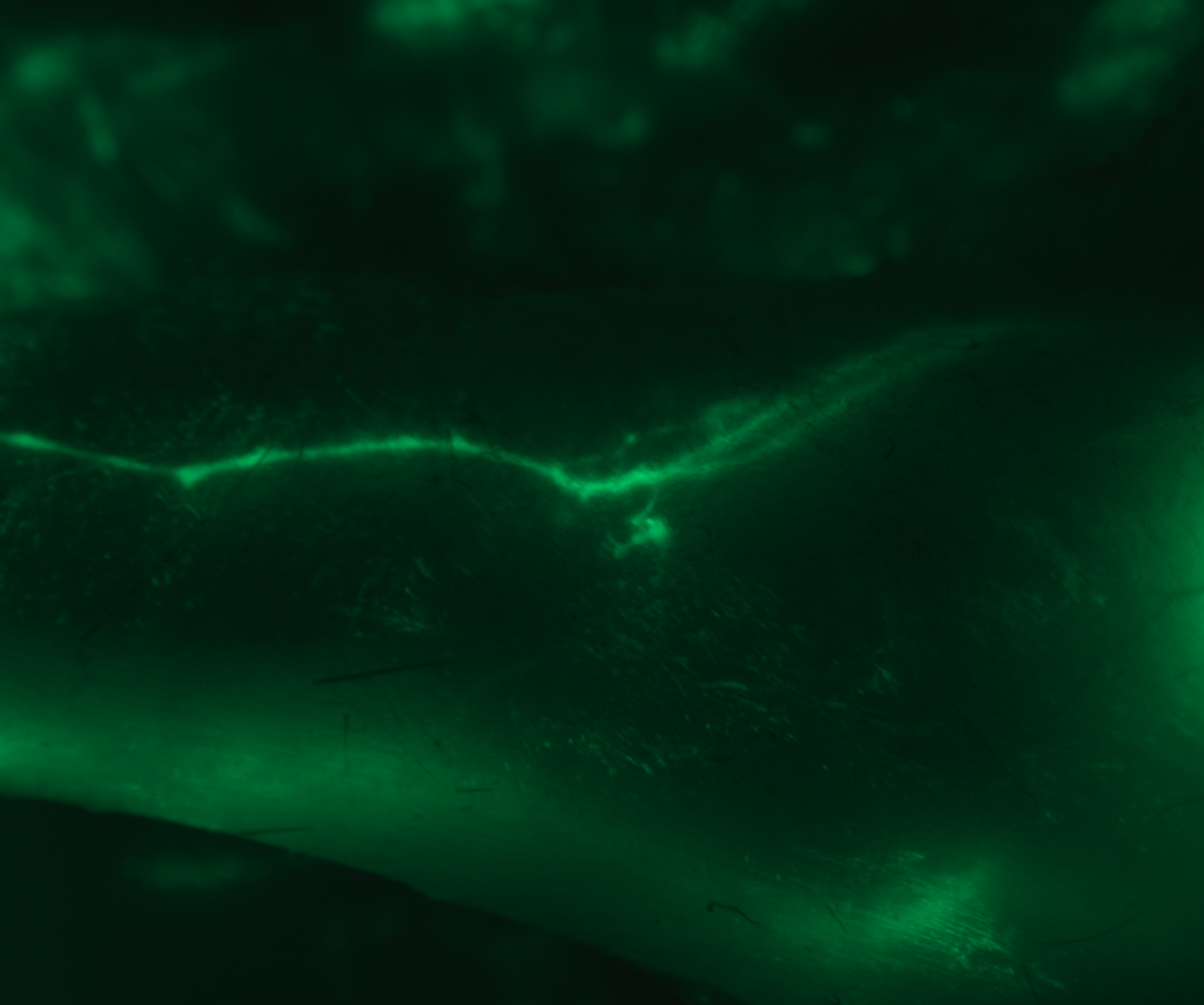

Supplement: Supplementary file 7 — Source Data Fig. 3 [file 44321_2023_17_MOESM7_ESM.zip › Figure 3/3C1.tif]

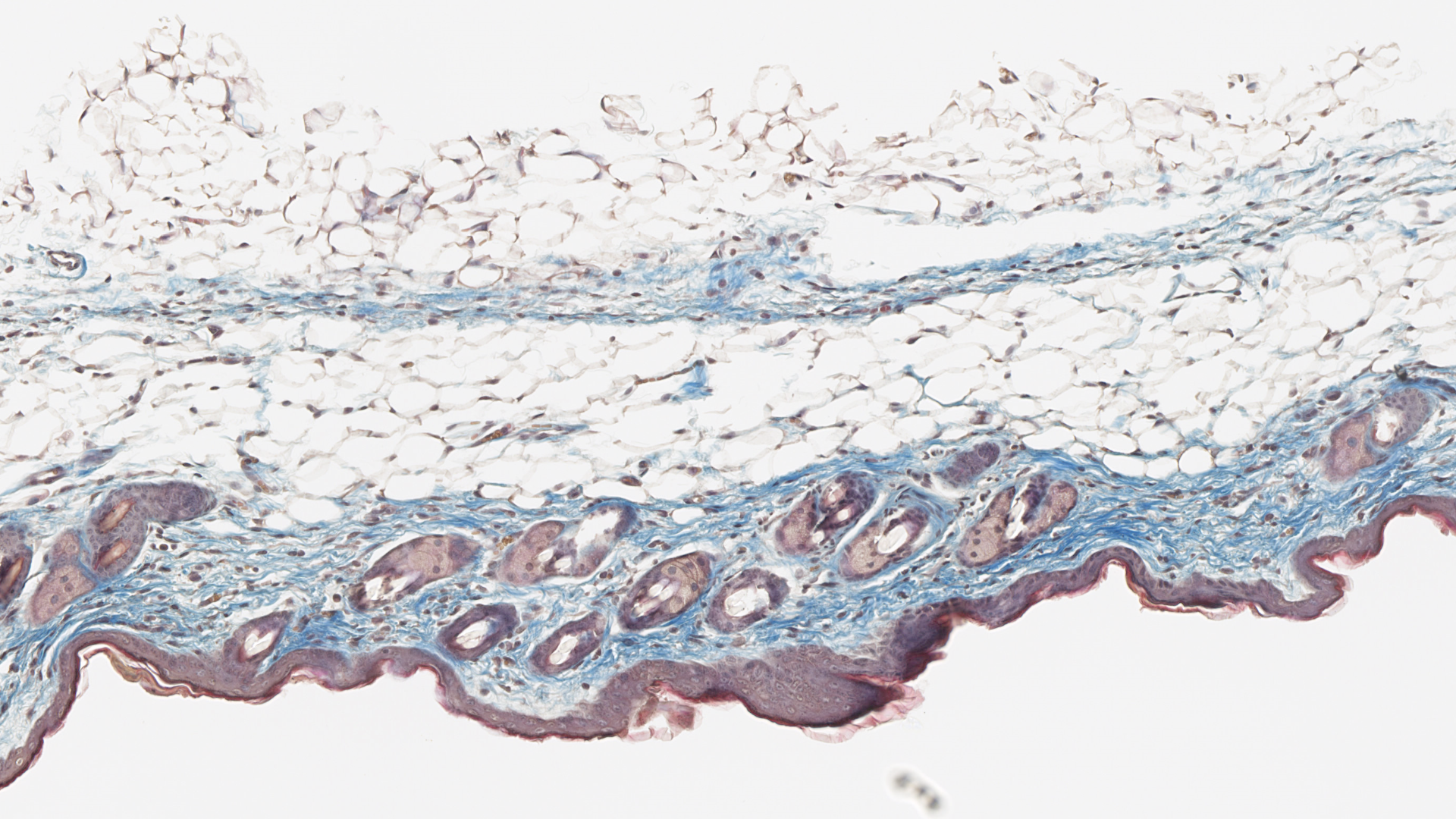

Supplement: Supplementary file 7 — Source Data Fig. 3 [file 44321_2023_17_MOESM7_ESM.zip › Figure 3/3D2.tif]

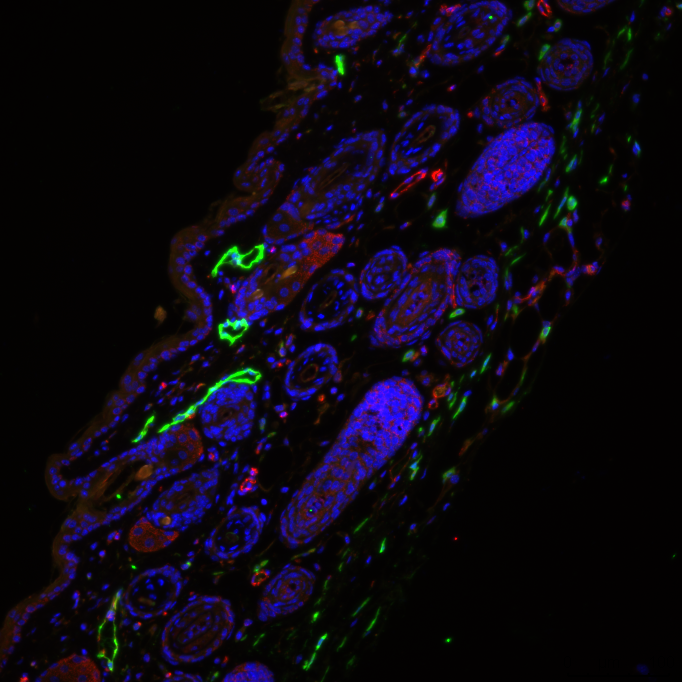

Supplement: Supplementary file 7 — Source Data Fig. 3 [file 44321_2023_17_MOESM7_ESM.zip › Figure 3/3I4.tif]

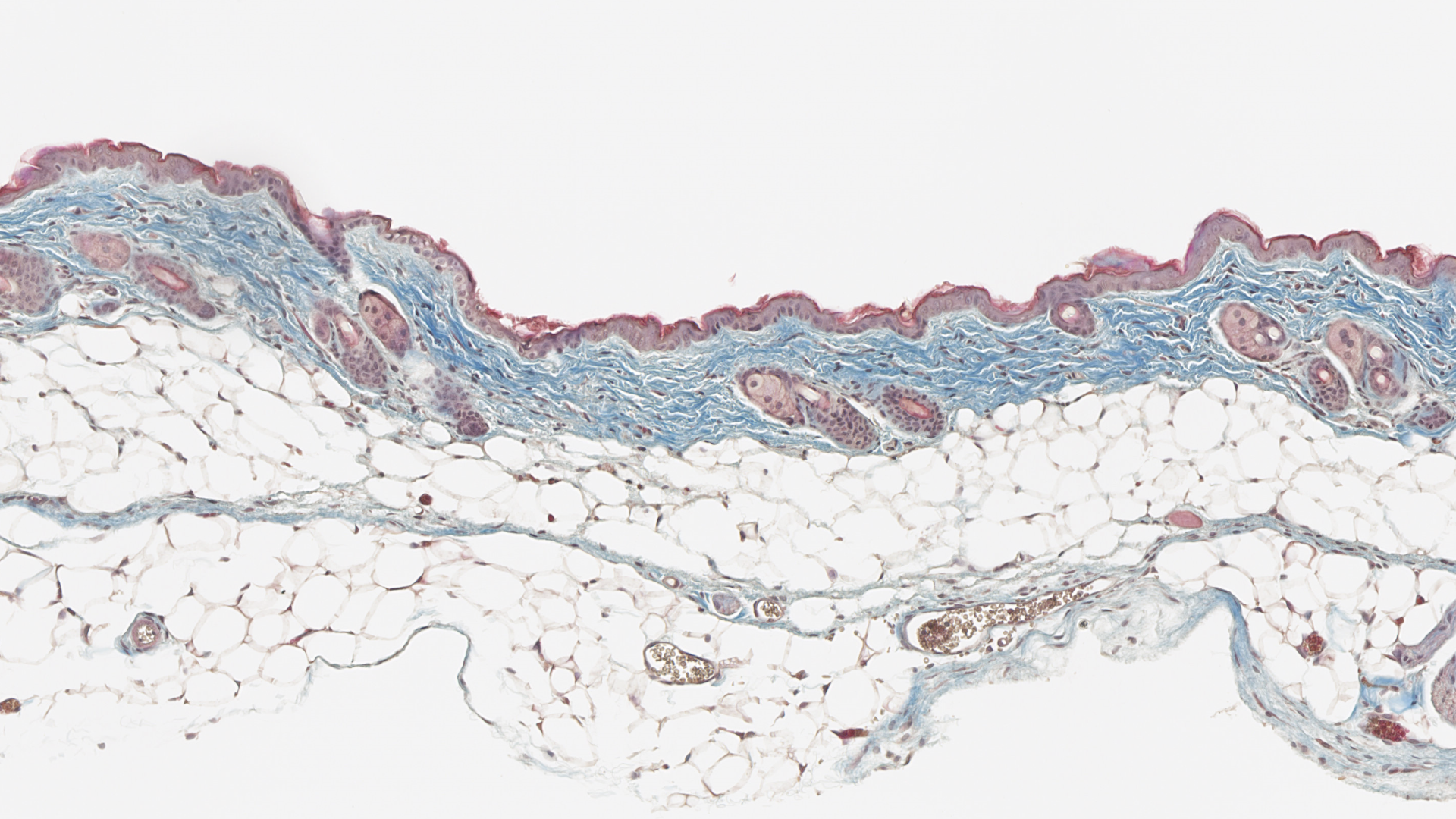

Supplement: Supplementary file 7 — Source Data Fig. 3 [file 44321_2023_17_MOESM7_ESM.zip › Figure 3/3D3.tif]

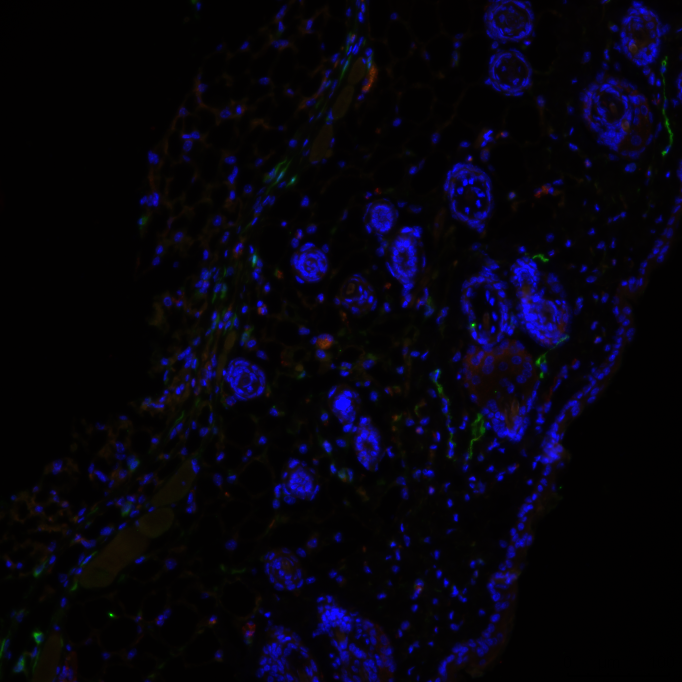

Supplement: Supplementary file 7 — Source Data Fig. 3 [file 44321_2023_17_MOESM7_ESM.zip › Figure 3/3I3.tif]

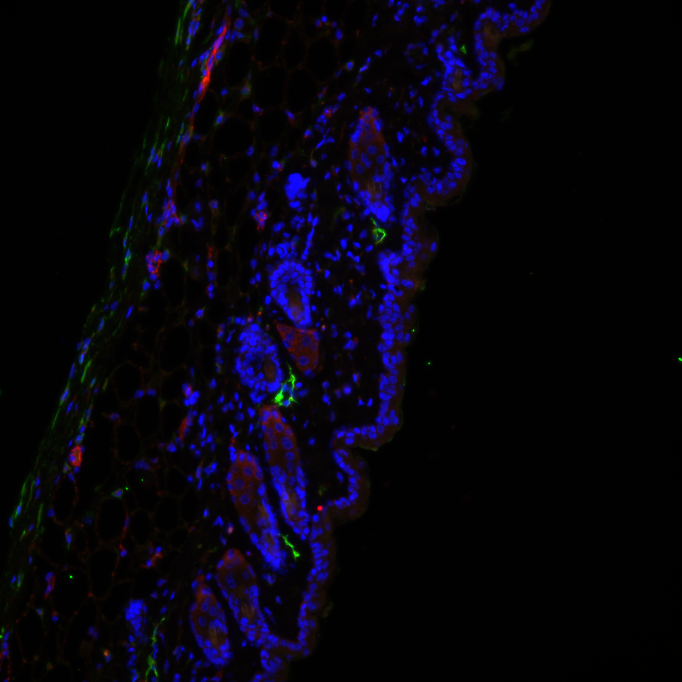

Supplement: Supplementary file 7 — Source Data Fig. 3 [file 44321_2023_17_MOESM7_ESM.zip › Figure 3/3I2.tif]

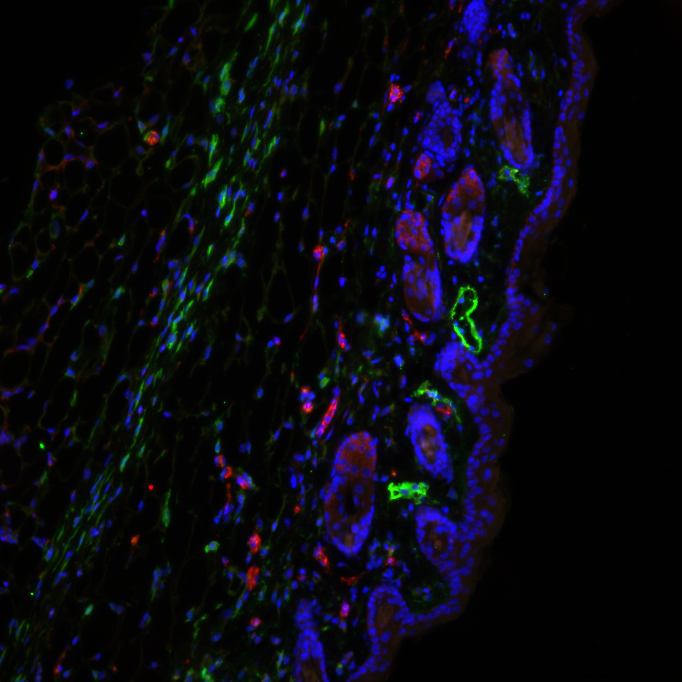

Supplement: Supplementary file 7 — Source Data Fig. 3 [file 44321_2023_17_MOESM7_ESM.zip › Figure 3/3I1.tif]

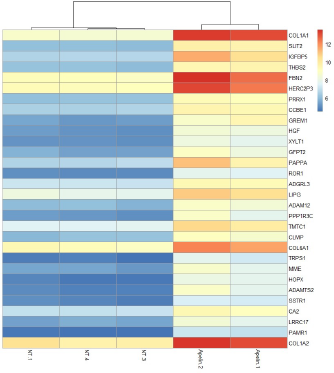

Supplement: Supplementary file 8 — Source Data Fig. 4 [file 44321_2023_17_MOESM8_ESM.zip › Figure 4/4B.tif]

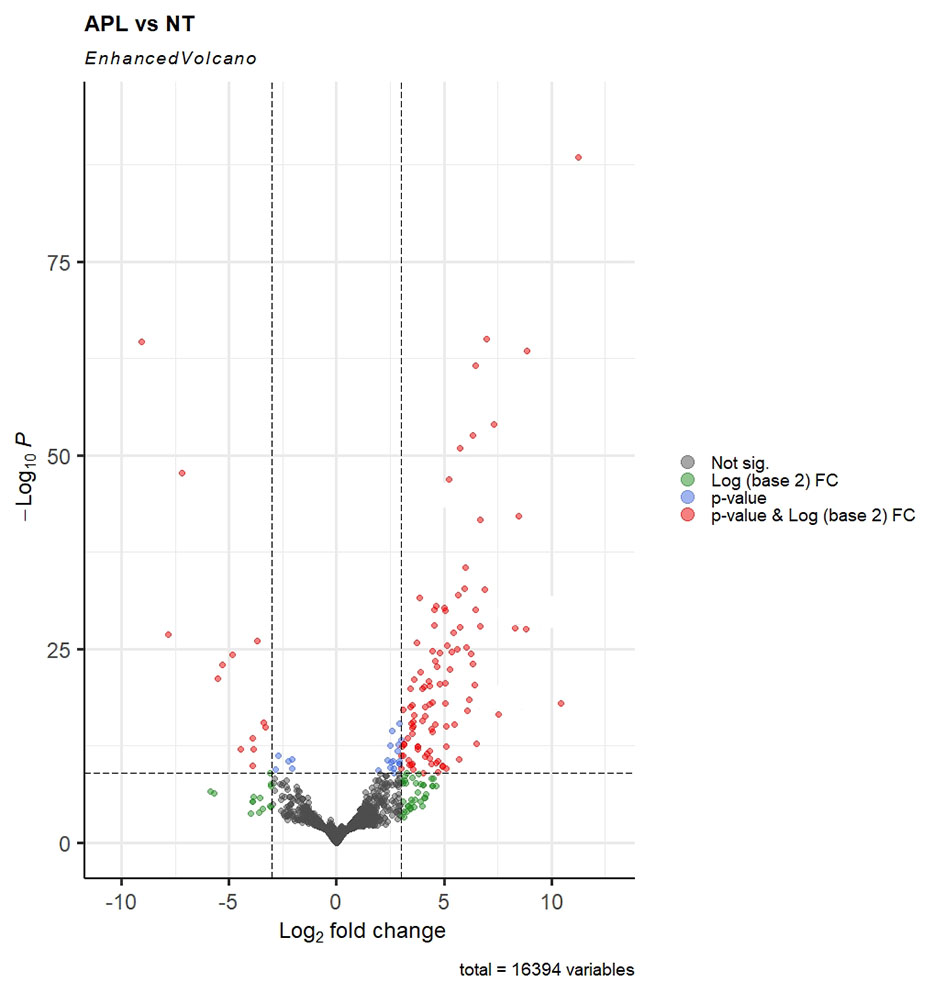

Supplement: Supplementary file 8 — Source Data Fig. 4 [file 44321_2023_17_MOESM8_ESM.zip › Figure 4/4A.jpg]

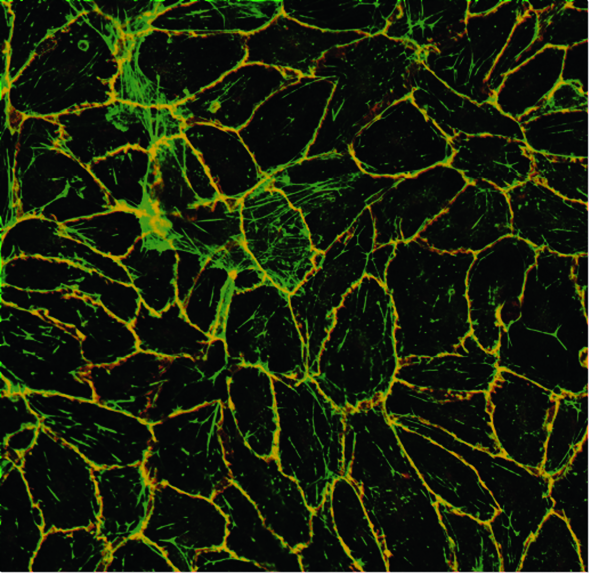

Supplement: Supplementary file 9 — Source Data Fig. 5 [file 44321_2023_17_MOESM9_ESM.zip › Figure 5/5G1.tif]

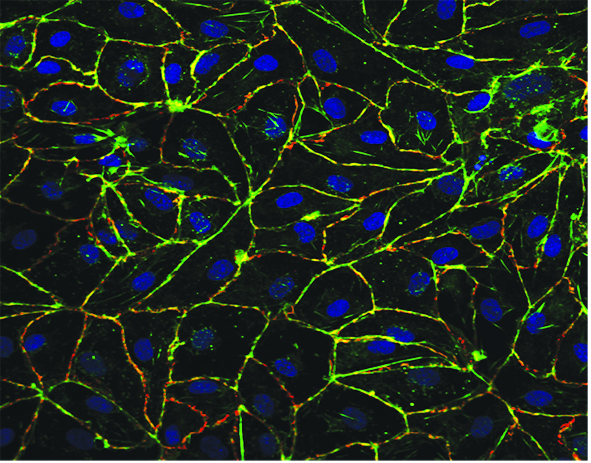

Supplement: Supplementary file 9 — Source Data Fig. 5 [file 44321_2023_17_MOESM9_ESM.zip › Figure 5/5G2.tif]

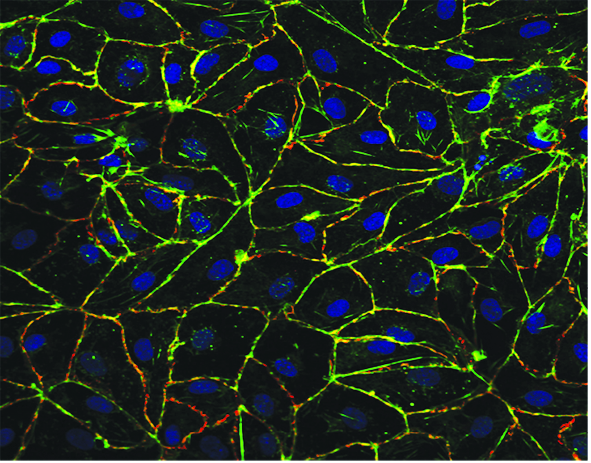

Supplement: Supplementary file 9 — Source Data Fig. 5 [file 44321_2023_17_MOESM9_ESM.zip › Figure 5/5G3.tif]

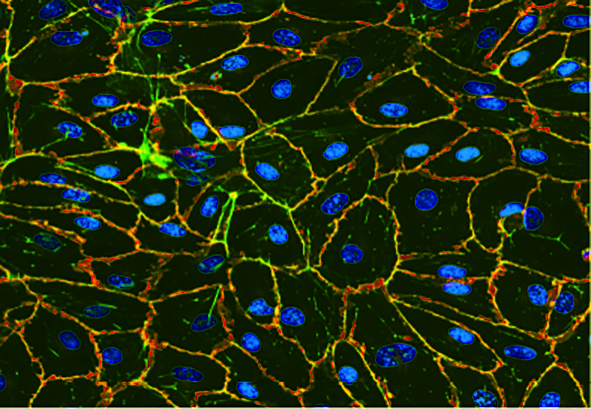

Supplement: Supplementary file 9 — Source Data Fig. 5 [file 44321_2023_17_MOESM9_ESM.zip › Figure 5/5G4.tif]

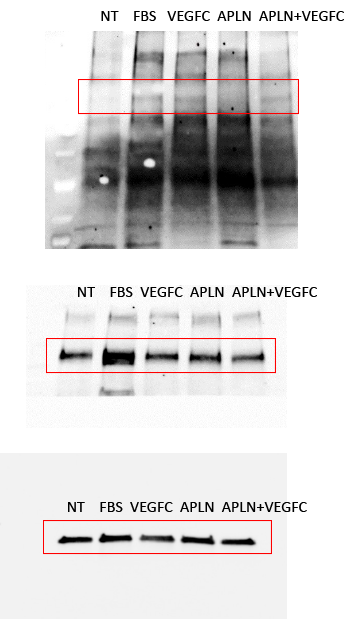

Supplement: Supplementary file 9 — Source Data Fig. 5 [file 44321_2023_17_MOESM9_ESM.zip › Figure 5/5H.tif]

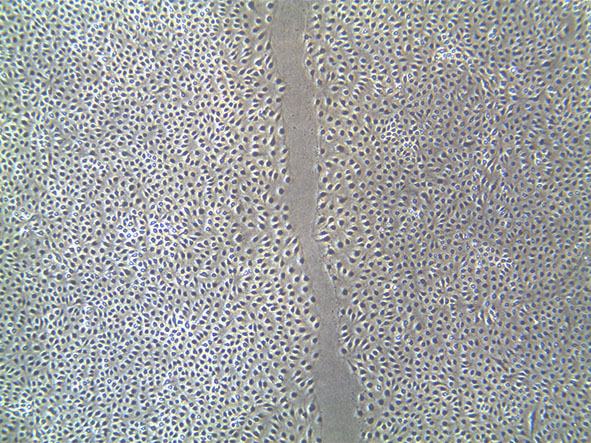

Supplement: Supplementary file 9 — Source Data Fig. 5 [file 44321_2023_17_MOESM9_ESM.zip › Figure 5/5E4.jpg]

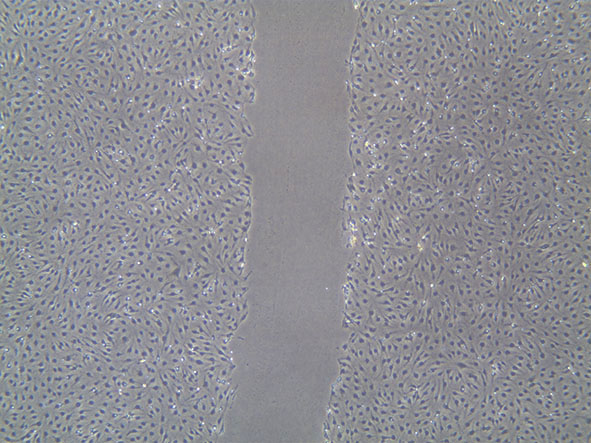

Supplement: Supplementary file 9 — Source Data Fig. 5 [file 44321_2023_17_MOESM9_ESM.zip › Figure 5/5E1.jpg]

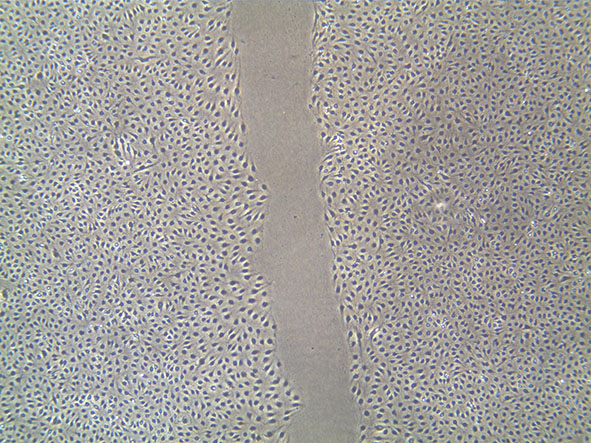

Supplement: Supplementary file 9 — Source Data Fig. 5 [file 44321_2023_17_MOESM9_ESM.zip › Figure 5/5E2.jpg]

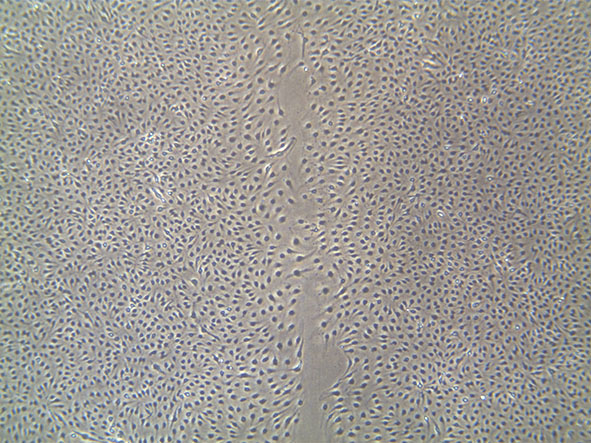

Supplement: Supplementary file 9 — Source Data Fig. 5 [file 44321_2023_17_MOESM9_ESM.zip › Figure 5/5E3.jpg]

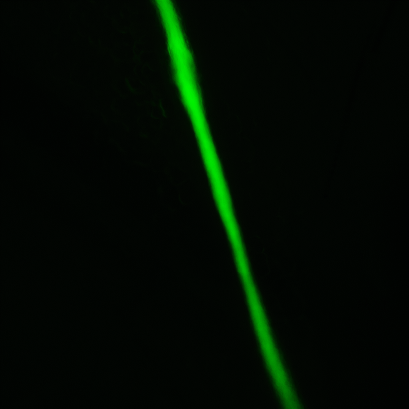

Supplement: Supplementary file 10 — Source Data Fig. 6 [file 44321_2023_17_MOESM10_ESM.zip › Figure 6/6A3.tif]

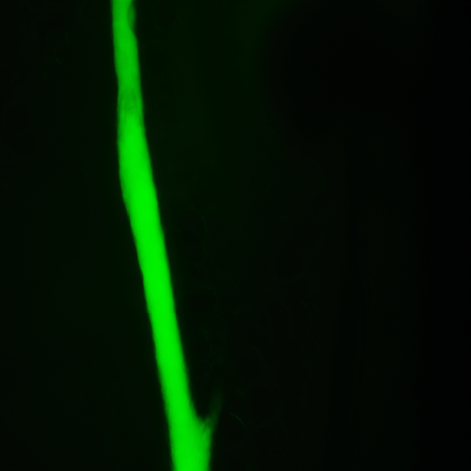

Supplement: Supplementary file 10 — Source Data Fig. 6 [file 44321_2023_17_MOESM10_ESM.zip › Figure 6/6A2.tif]

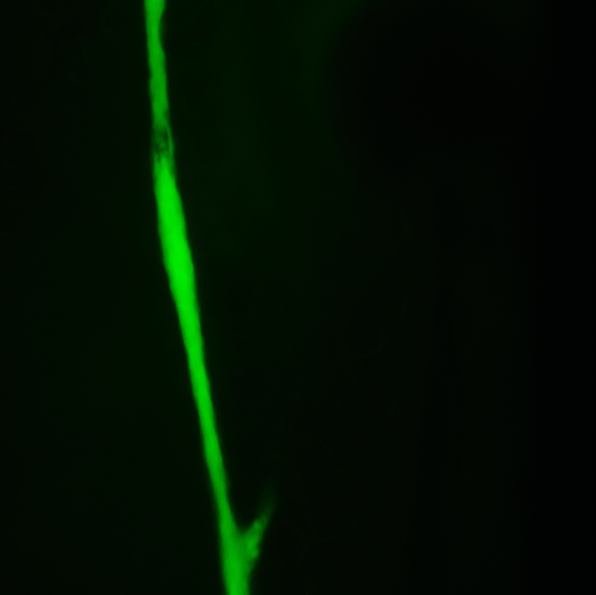

Supplement: Supplementary file 10 — Source Data Fig. 6 [file 44321_2023_17_MOESM10_ESM.zip › Figure 6/6A1.tif]

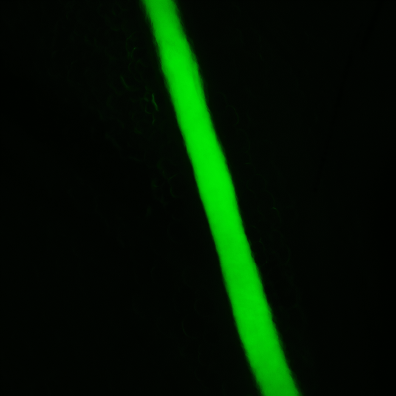

Supplement: Supplementary file 10 — Source Data Fig. 6 [file 44321_2023_17_MOESM10_ESM.zip › Figure 6/6A4.tif]

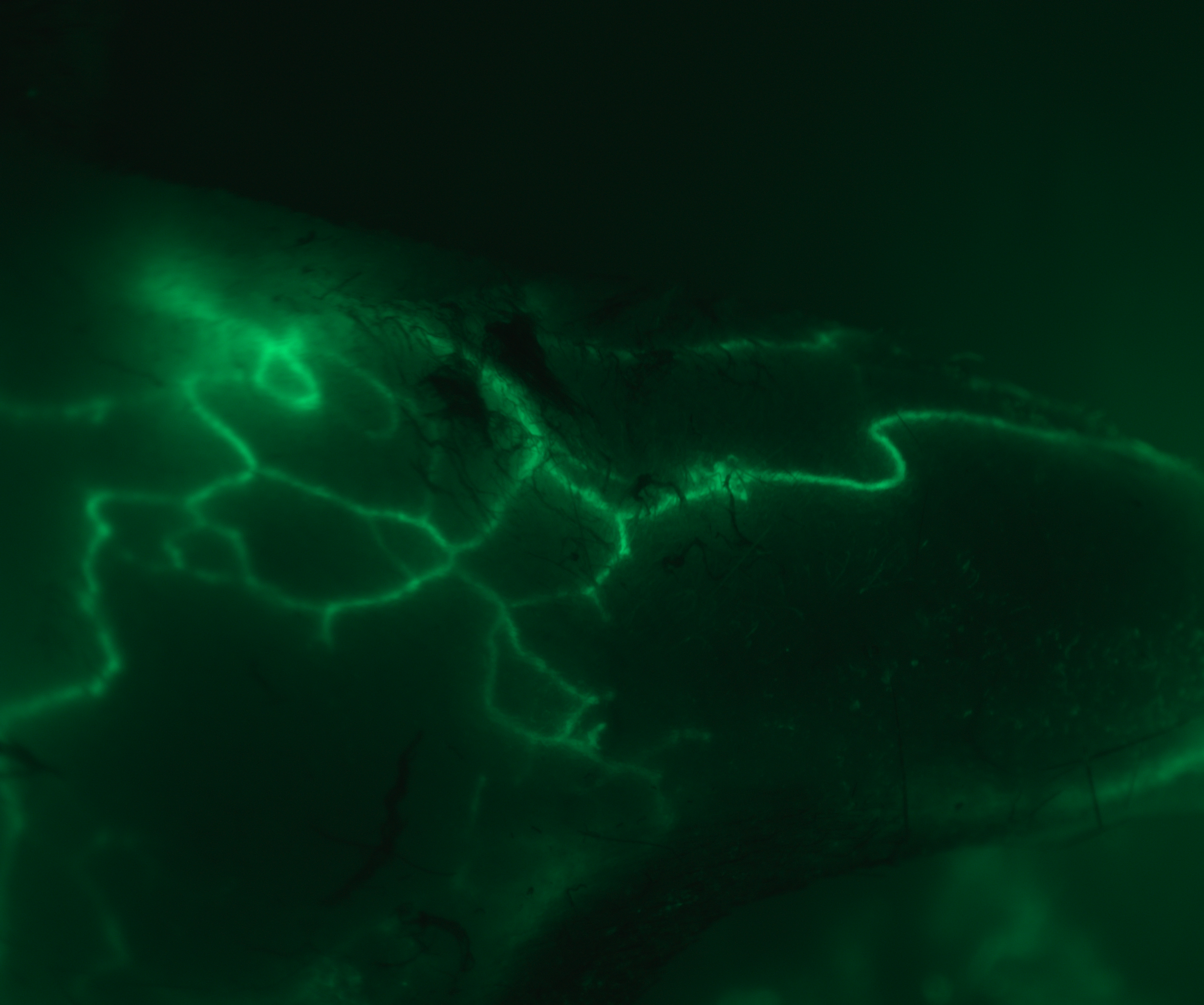

Supplement: Supplementary file 10 — Source Data Fig. 6 [file 44321_2023_17_MOESM10_ESM.zip › Figure 6/6F4.tif]

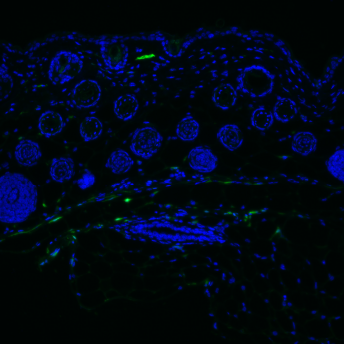

Supplement: Supplementary file 10 — Source Data Fig. 6 [file 44321_2023_17_MOESM10_ESM.zip › Figure 6/6G1.tif]

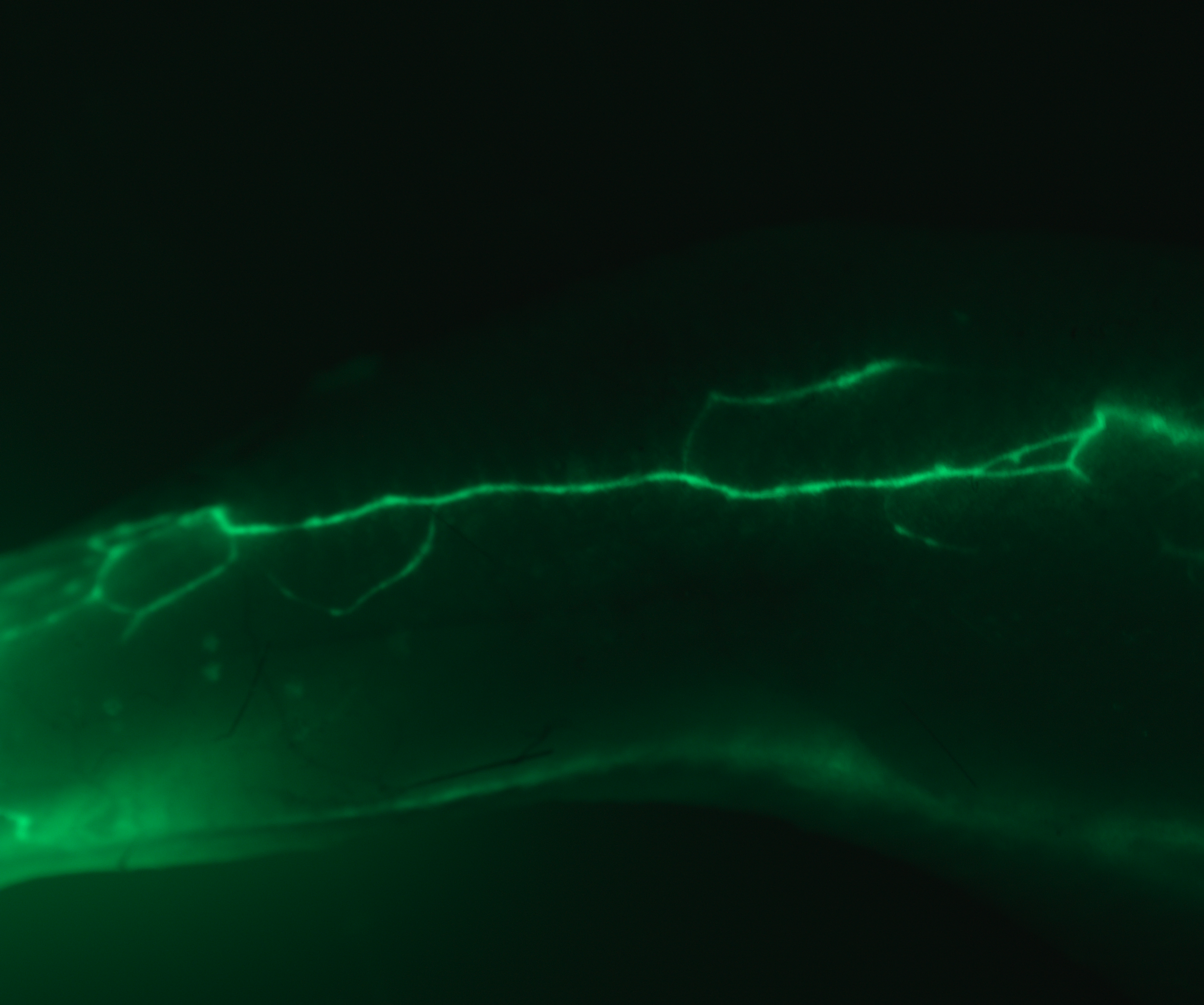

Supplement: Supplementary file 10 — Source Data Fig. 6 [file 44321_2023_17_MOESM10_ESM.zip › Figure 6/6F5.tif]

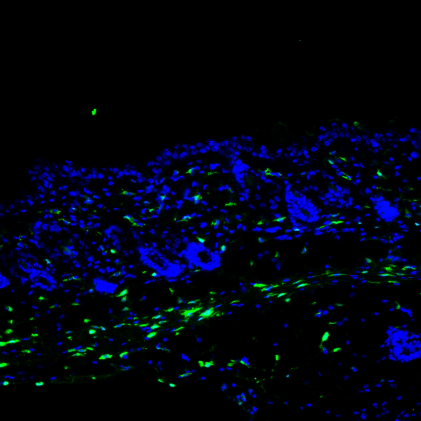

Supplement: Supplementary file 10 — Source Data Fig. 6 [file 44321_2023_17_MOESM10_ESM.zip › Figure 6/6G3.tif]

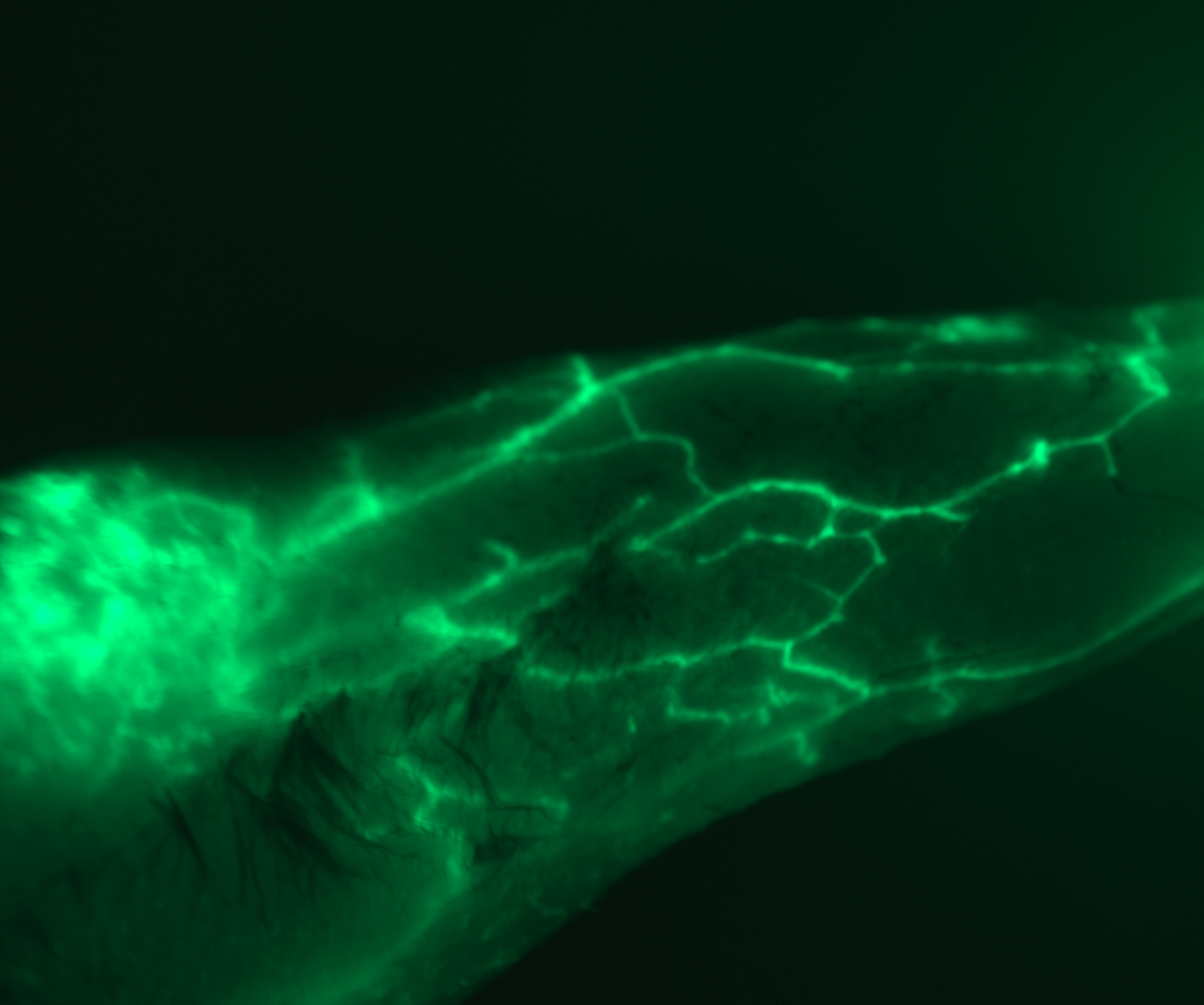

Supplement: Supplementary file 10 — Source Data Fig. 6 [file 44321_2023_17_MOESM10_ESM.zip › Figure 6/6F6.tif]

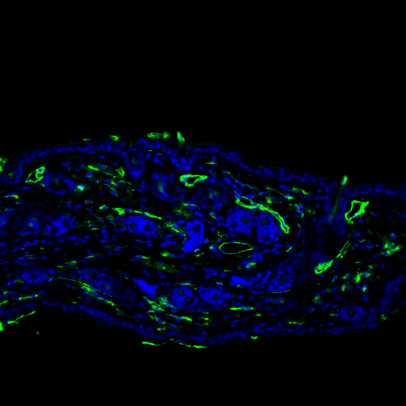

Supplement: Supplementary file 10 — Source Data Fig. 6 [file 44321_2023_17_MOESM10_ESM.zip › Figure 6/6G2.tif]

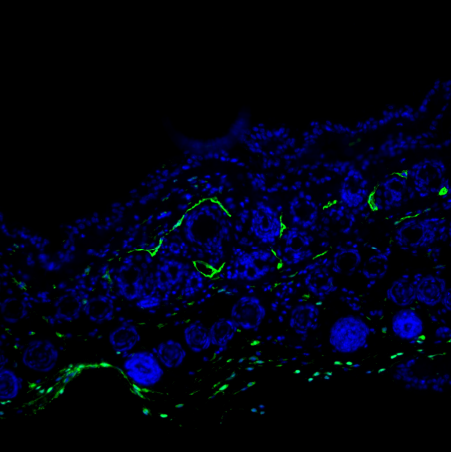

Supplement: Supplementary file 10 — Source Data Fig. 6 [file 44321_2023_17_MOESM10_ESM.zip › Figure 6/6G6.tif]

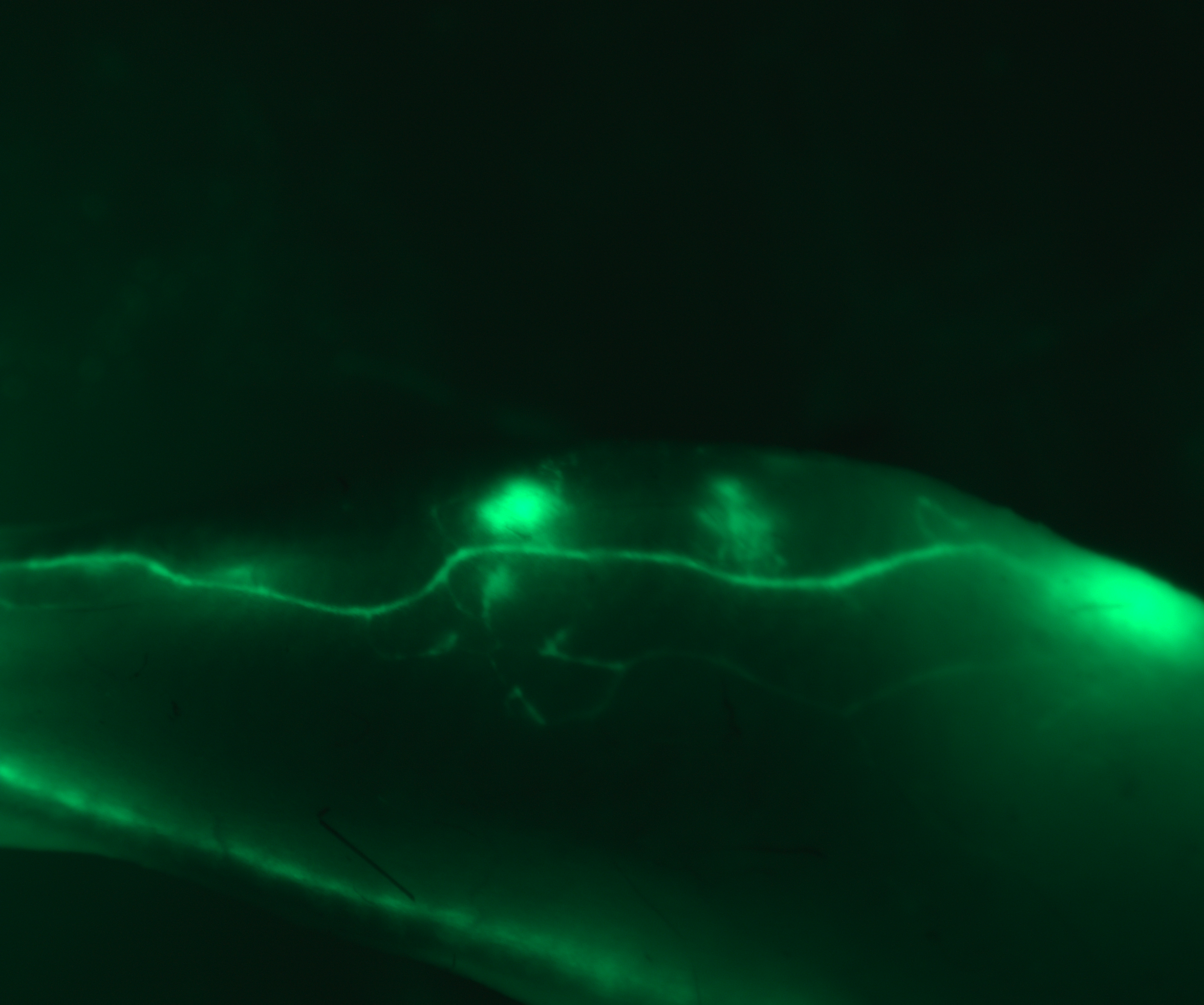

Supplement: Supplementary file 10 — Source Data Fig. 6 [file 44321_2023_17_MOESM10_ESM.zip › Figure 6/6F2.tif]

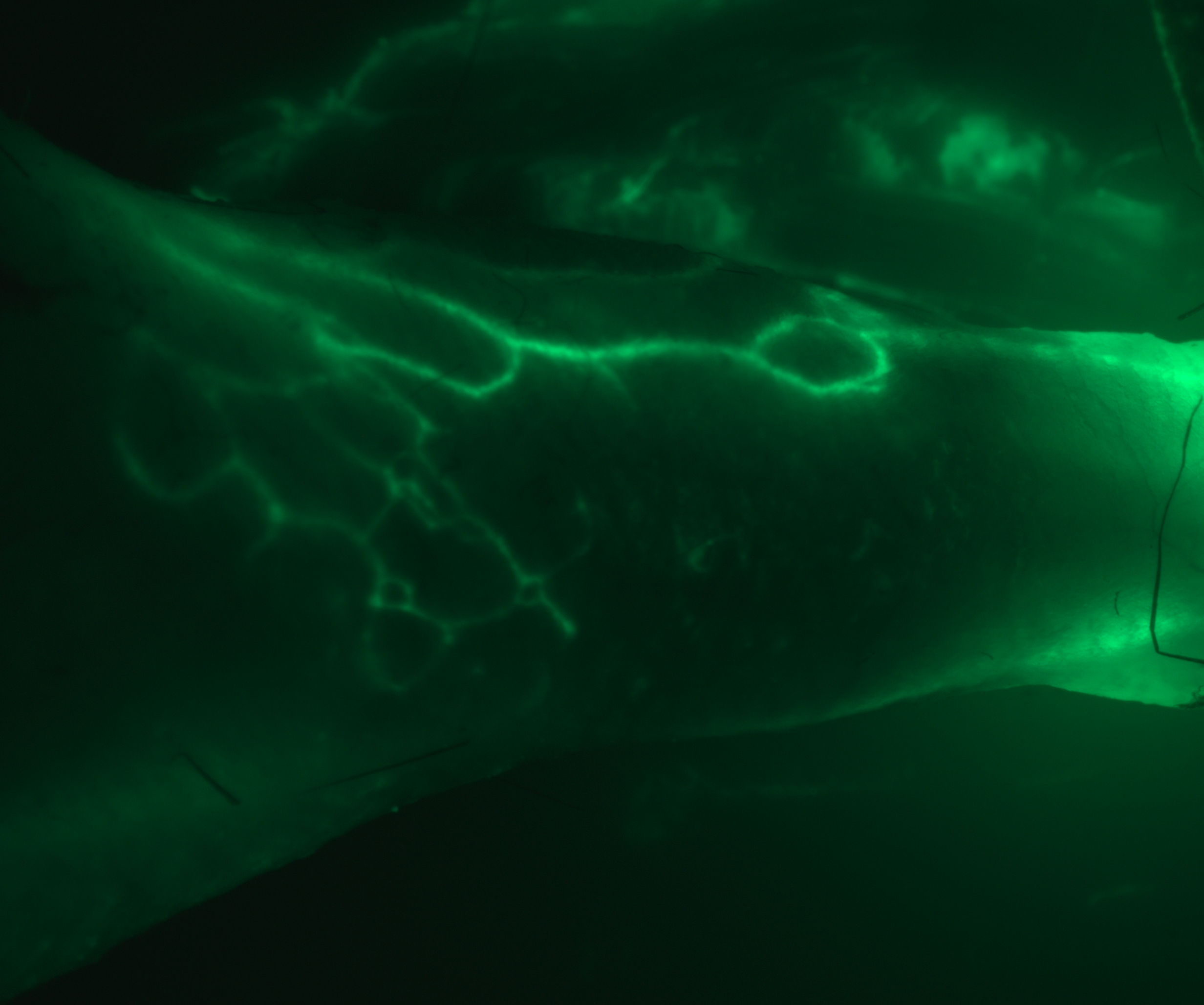

Supplement: Supplementary file 10 — Source Data Fig. 6 [file 44321_2023_17_MOESM10_ESM.zip › Figure 6/6F3.tif]

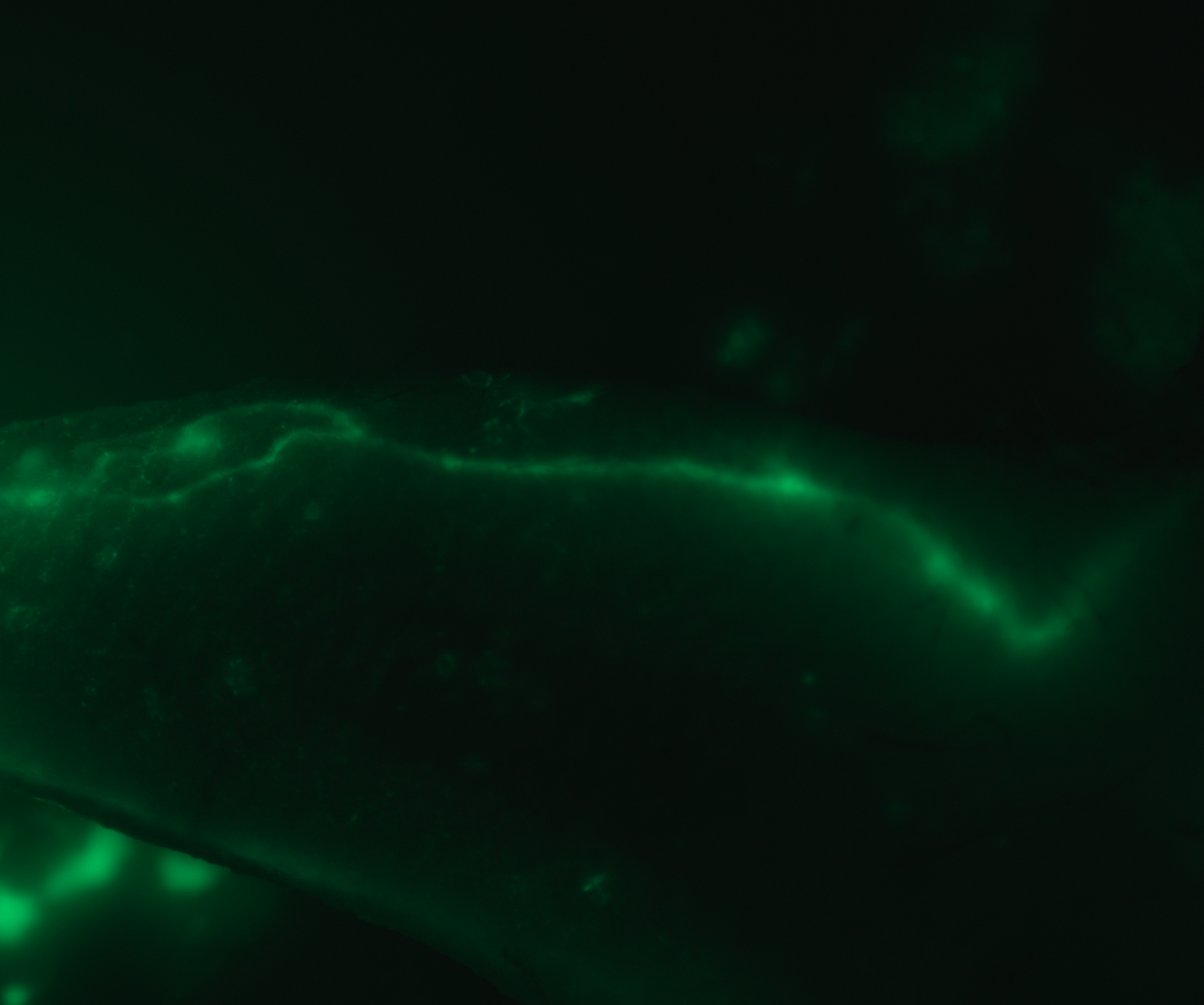

Supplement: Supplementary file 10 — Source Data Fig. 6 [file 44321_2023_17_MOESM10_ESM.zip › Figure 6/6F1.tif]

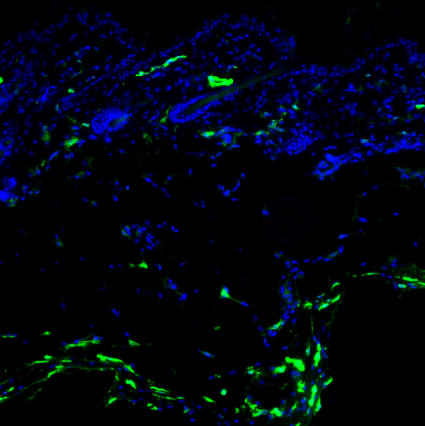

Supplement: Supplementary file 10 — Source Data Fig. 6 [file 44321_2023_17_MOESM10_ESM.zip › Figure 6/6G5.tif]

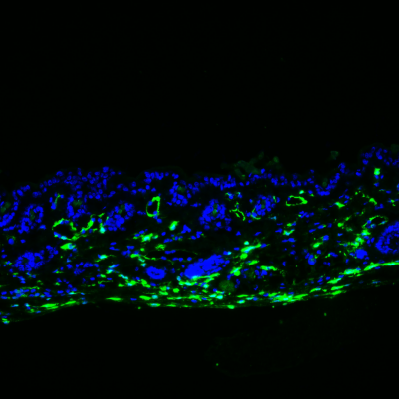

Supplement: Supplementary file 10 — Source Data Fig. 6 [file 44321_2023_17_MOESM10_ESM.zip › Figure 6/6G4.tif]

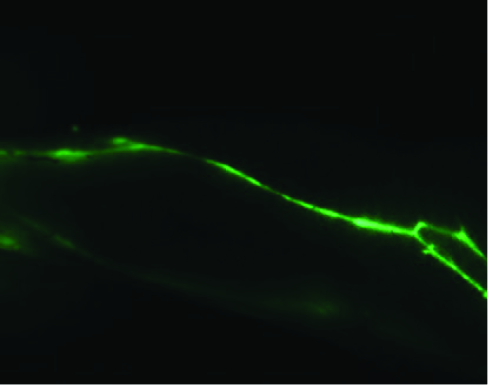

Supplement: Supplementary file 12 — Source Data Fig. 8 [file 44321_2023_17_MOESM12_ESM.zip › Figure 8/8H1.tif]

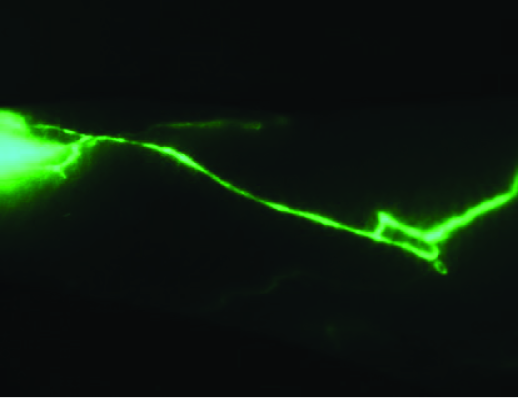

Supplement: Supplementary file 12 — Source Data Fig. 8 [file 44321_2023_17_MOESM12_ESM.zip › Figure 8/8H3.tif]

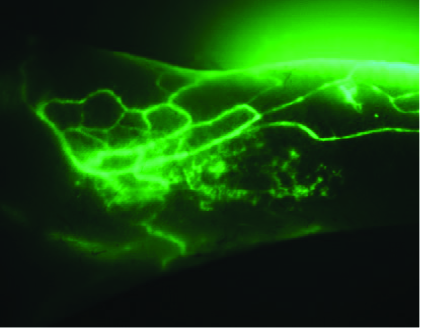

Supplement: Supplementary file 12 — Source Data Fig. 8 [file 44321_2023_17_MOESM12_ESM.zip › Figure 8/8H2.tif]

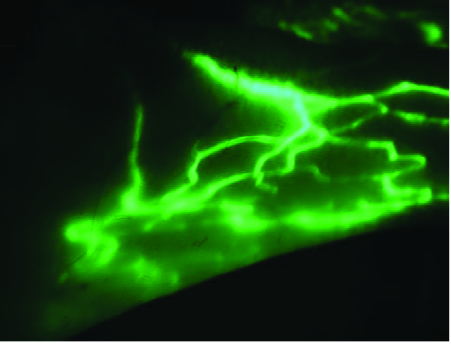

Supplement: Supplementary file 12 — Source Data Fig. 8 [file 44321_2023_17_MOESM12_ESM.zip › Figure 8/8H6.tif]

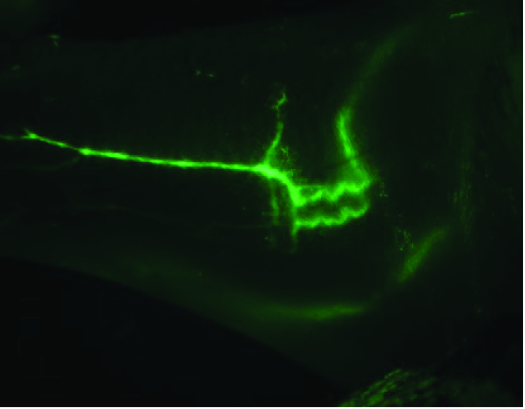

Supplement: Supplementary file 12 — Source Data Fig. 8 [file 44321_2023_17_MOESM12_ESM.zip › Figure 8/8H7.tif]

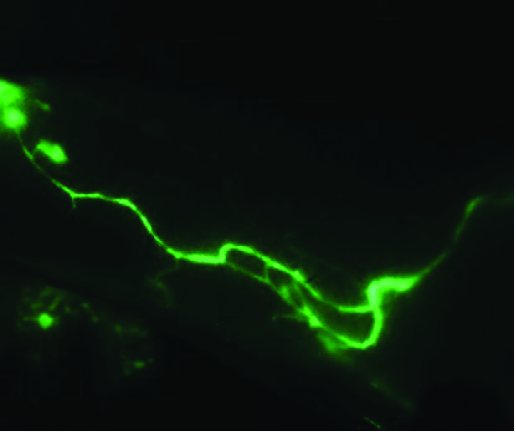

Supplement: Supplementary file 12 — Source Data Fig. 8 [file 44321_2023_17_MOESM12_ESM.zip › Figure 8/8H5.tif]

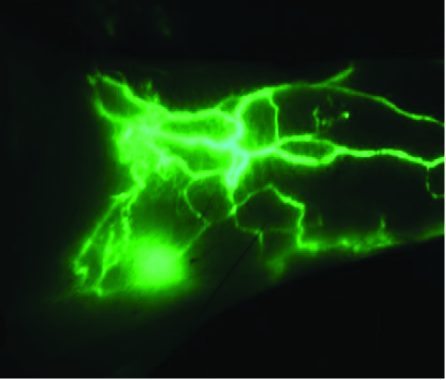

Supplement: Supplementary file 12 — Source Data Fig. 8 [file 44321_2023_17_MOESM12_ESM.zip › Figure 8/8H4.tif]

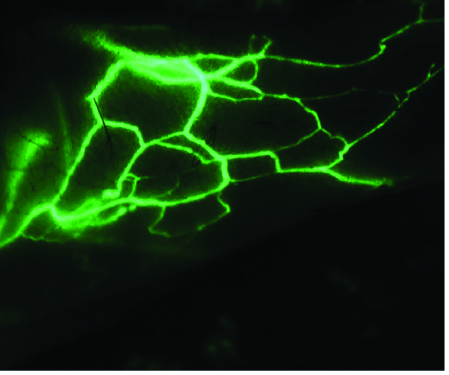

Supplement: Supplementary file 12 — Source Data Fig. 8 [file 44321_2023_17_MOESM12_ESM.zip › Figure 8/8H8.tif]
