# Supplementary material for: Apelin-VEGF-C mRNA delivery as therapeutic for the treatment of secondary lymphedema
Source: EMBO Mol Med. 2024 Jan 2;16(2):386–415. doi: 10.1038/s44321-023-00017-7 (PMC10898257; doi:10.1038/s44321-023-00017-7)
Supplement: Supplementary file 13 — Expanded View Figures [file 44321_2023_17_MOESM13_ESM.pdf]

Expanded View Figures

Figure EV1. Effect of APLN vector on skin angiogenesis.

(**A**) Skin section staining with Lyve1 (green) and APJ (red). Scale bar = 50 μm. (**B**) Skin section staining with vimentin (green) and APJ (red). Scale bar = 50 μm. (**C**) Skin section staining with CD68 (green) and APJ (red). Scale bar = 50 μm. (**D**) APLN mRNA expression in skin from ctrl and LD limb. Data represent mean ± SEM ($n = 3$) ($^*P < 0.05$, unpaired t test). (**E**) Skin sections of control or LD limb from LV-APLN-treated mice were stained for CD31 (red). DNA was stained with DAPI. Scale bar = 100 μm. (**F**) Quantification of blood vessels per field according the limb and treatment of mice. Data represent mean ± SEM ($n = 9$ control and $n = 8$ APLN-treated mice) ($^*P < 0.05$, $^{**}P < 0.01$, one-way ANOVA). (**G**) Vascular permeability assay by Evans blue extravasation. Left ear intradermally injected with 2 μL of control lentivector, right ear injected with 2 μL of APLN lentivector. (**H**) Quantification of extravasated Evans blue dye. Data represent mean ± SEM ($^*P < 0.05$, unpaired t test).

© The Author(s)

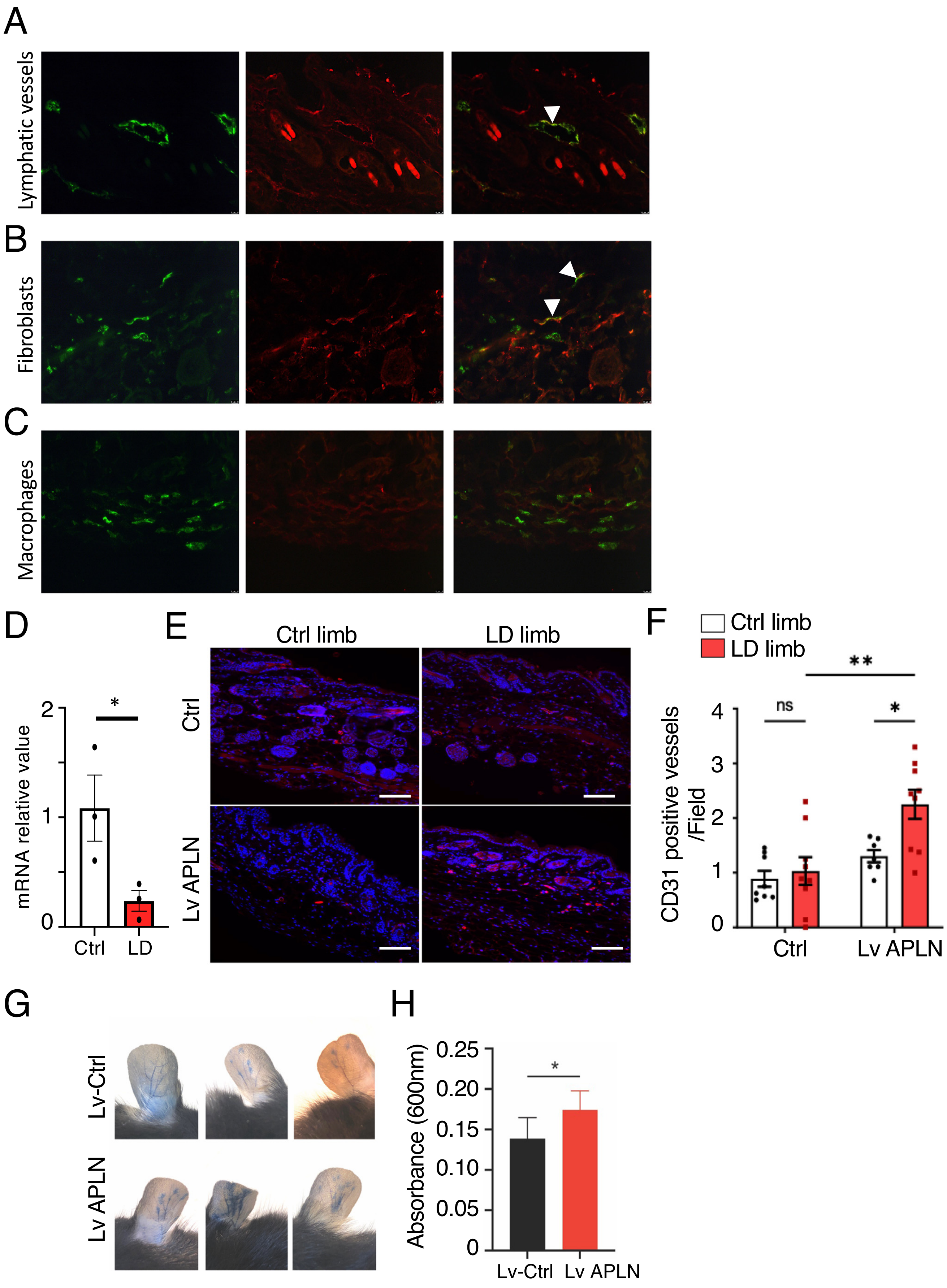
A
Lymphatic vessels
B
Fibroblasts
C
Macrophages
D
mRNA relative value
*
2
1
0
Ctrl
LD
E
Ctrl limb
LD limb
Ctrl
Lv APLN
F
Ctrl limb
LD limb
**
ns
*
CD31 positive vessels /Field
4
3
2
1
0
Ctrl
Lv APLN
G
Lv-Ctrl
Lv APLN
H
Absorbance (600nm)
*
0.25
0.20
0.15
0.10
0.05
0
Lv-Ctrl
Lv APLN

 © The Author(s)

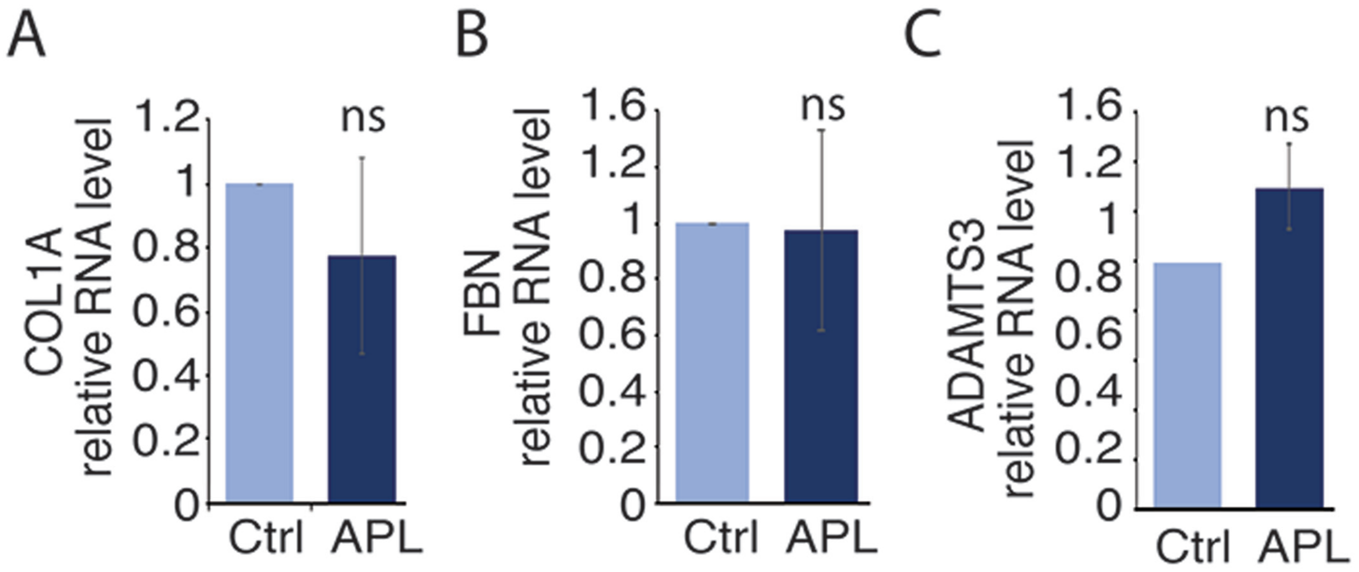

Figure EV2. Gene expression in APLN-stimulated HDLEC.

(**A–C**). RT-qPCR showing the expression of COL1A (**A**), FBN (**B**), and ADAMTS3 (**C**) in APLN-stimulated HDLEC. Data represent mean ± SEM (ns=non significant, unpaired *t* test).

© The Author(s)

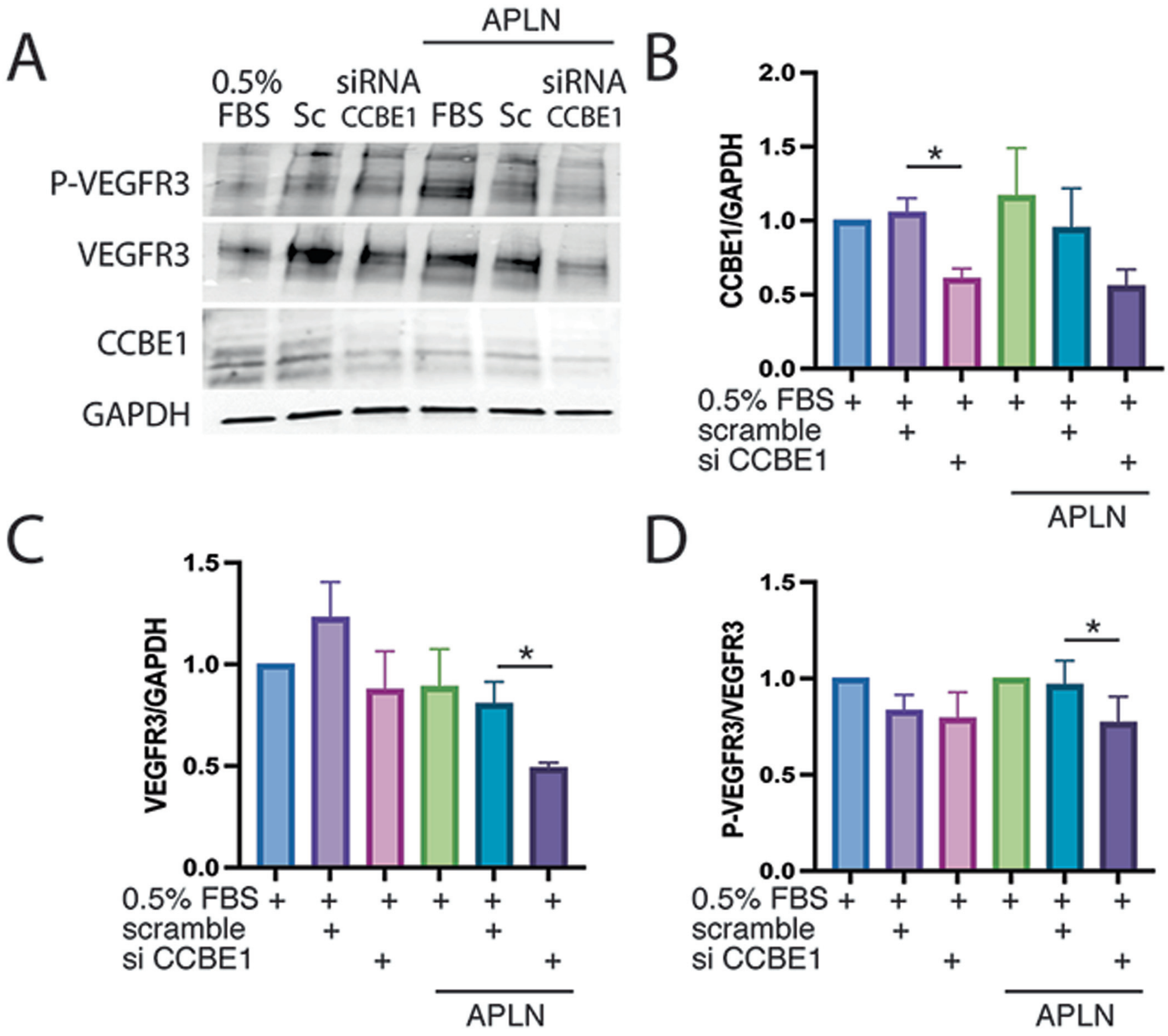

Figure EV3. Stimulation of VEGFR3 phosphorylation by APLN.

(**A**) Representatives phospho-VEGFR3/VEGFR3 and CCBE1 immunoblots of HDLEC treated with APLN +/− siRNA CCBE1. (**B**) Graphs represent quantification of CCBE1/GAPDH protein ratio from at least three independent experiments. Data represent mean ± SEM ($n = 3$ independent replicates) (*$P < 0.05$, two-way ANOVA). (**C**) Graphs represent quantification of VEGFR3/GAPDH protein ratio from at least three independent experiments. Data represent mean ± SEM ($n = 3$ independent replicates) (*$P < 0.05$, two-way ANOVA). (**D**) Graphs represent quantification of phospho/total protein ratio of VEGFR3 from at least three independent experiments. Data represent mean ± SEM ($n = 3$ independent replicates) (*$P < 0.05$, two-way ANOVA).

 © The Author(s)

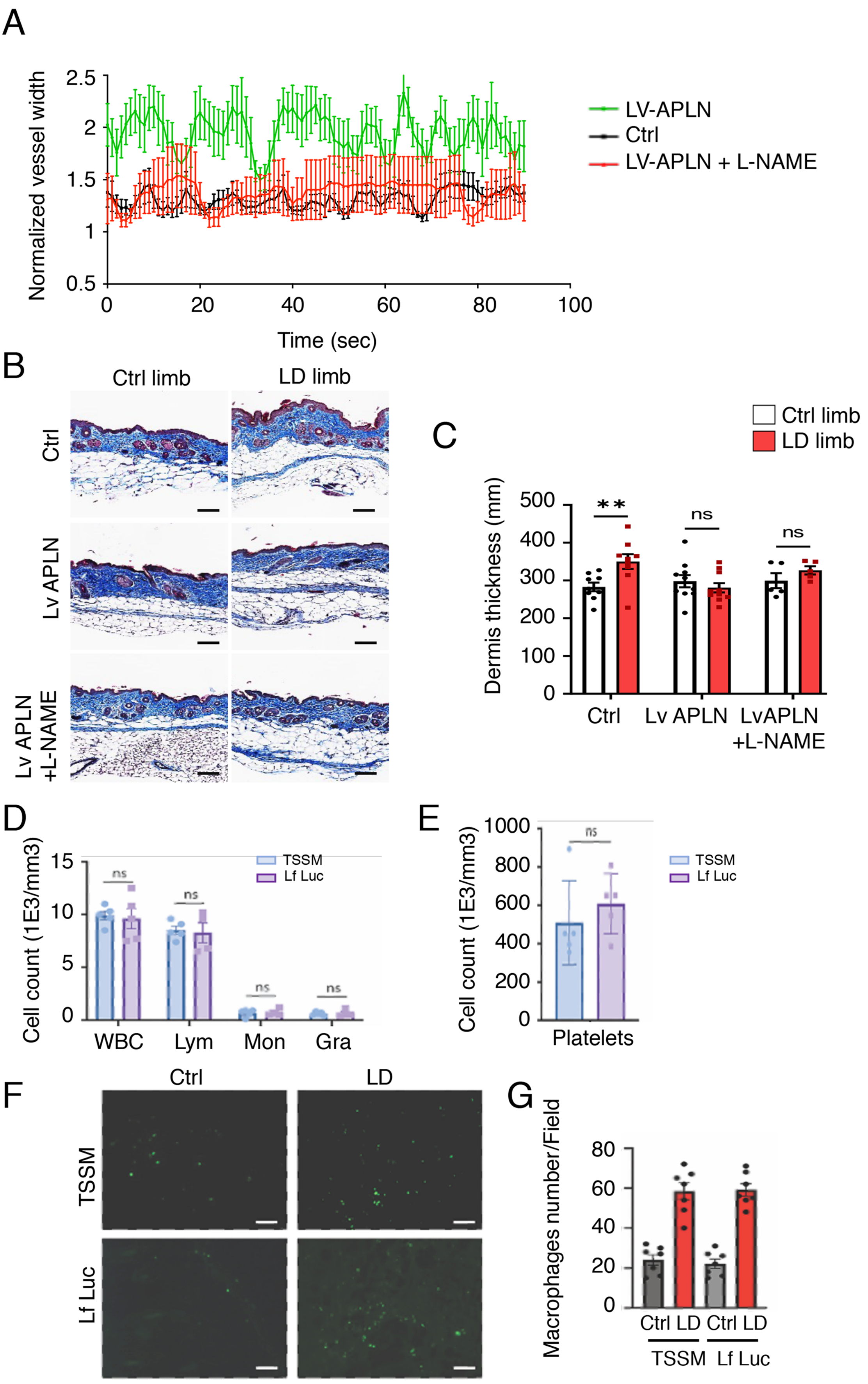
A
Normalized vessel width
2.5
2
1.5
1
0.5
0
20
40
60
80
100
Time (sec)
LV-APLN
Ctrl
LV-APLN + L-NAME
B
Ctrl limb
LD limb
Ctrl
Lv APLN
Lv APLN +L-NAME
C
Ctrl limb
LD limb
Dermis thickness (mm)
500
400
300
200
100
0
**
ns
ns
Ctrl
Lv APLN
LvAPLN +L-NAME
D
Cell count (1E3/mm3)
15
10
5
0
TSSM
Lf Luc
WBC
Lym
Mon
Gra
E
Cell count (1E3/mm3)
1000
800
600
400
200
0
Platelets
F
Ctrl
LD
TSSM
Lf Luc
G
Macrophages number/Field
80
60
40
20
0
Ctrl LD Ctrl LD
TSSM Lf Luc

Figure EV4. Effect of L-NAME on dermis fibrosis.

(**A**) Graph represent mean vessel width normalized to the minimum vessel width. (LV-APLN $n = 8$, Ctrl $n = 7$ and LV-APLN + L-NAME $n = 4$). (**B**) Fibrosis was evaluated by Masson's trichrome staining of the skin in control mice ($n = 9$) treated with APLN lentivector ($n = 9$) and LV-APLN + L-NAME ($n = 5$). (scale bar: 50 μm). (**C**) graph display the quantification of dermis thickness. Data represent mean ± SEM ($n = 9$) (**$P < 0.01$, two-way ANOVA). (**D**) Number of total circulating white blood cells, lymphocytes, monocytes and granulocytes in blood from mice 2 weeks after Lentiflash-APLN intradermal injection. Data represent mean ± SEM ($n = 5$) (ns = non significant, unpaired *t* test). (**E**) Number of blood platelet cells from mice 2 weeks after Lentiflash-APLN intradermal injection. Data represent mean ± SEM ($n = 5$) (ns=non significant, unpaired *t* test). (**F**) Representative images of macrophages (F4/80) immunodetection at the site of injection in skin from mice injected with Lf (Lf Luc) or adjuvant (TSSM) (scale bar = 50 μm). (**G**) Quantification of the number of macrophages at the site of Lf injection or TSSM control. Data represent mean ± SEM ($n = 7$).

 © The Author(s)

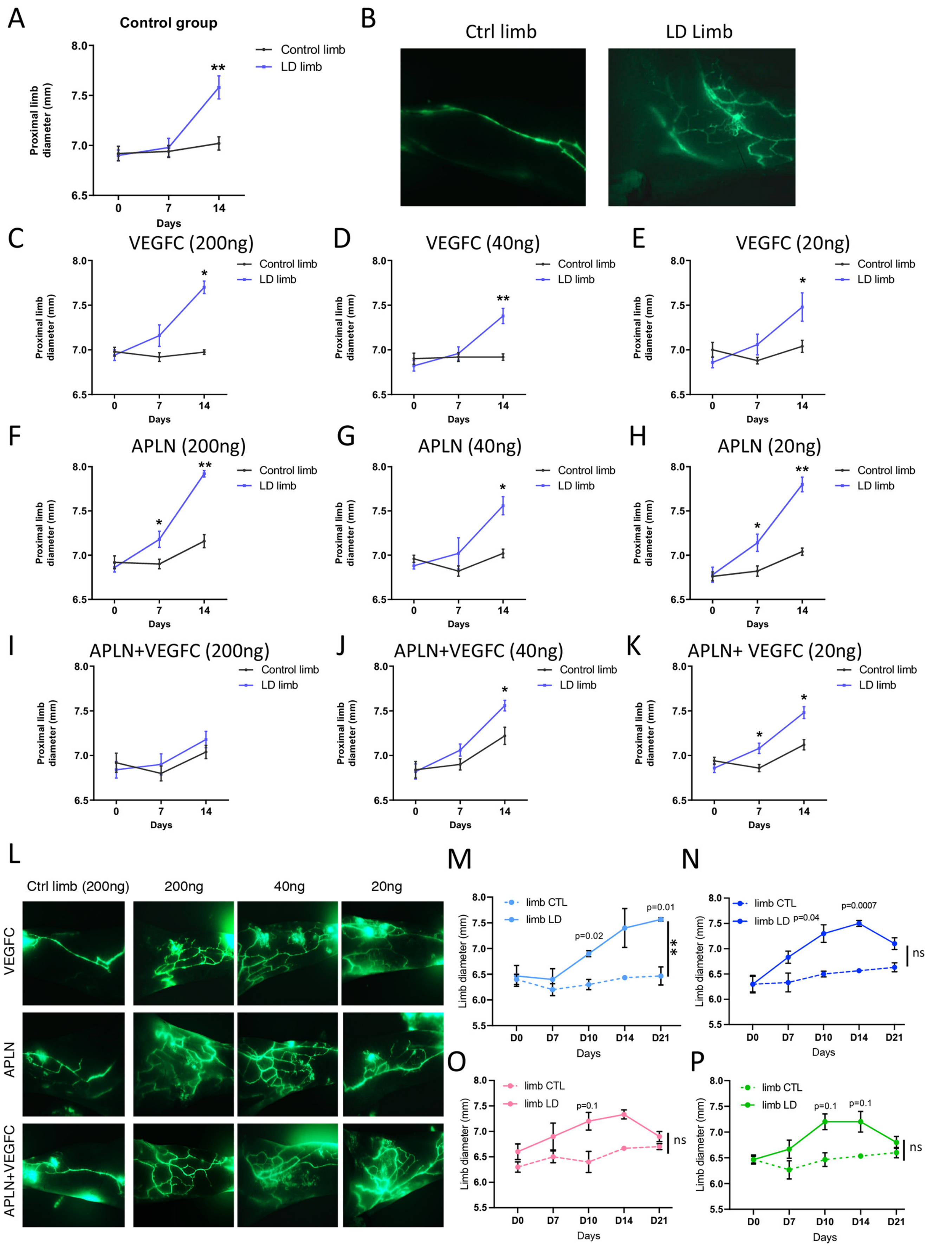
A
Control group
Control limb
LD limb
Proximal limb diameter (mm)
Days
B
Ctrl limb
LD Limb
C
VEGFC (200ng)
D
VEGFC (40ng)
E
VEGFC (20ng)
F
APLN (200ng)
G
APLN (40ng)
H
APLN (20ng)
I
APLN+VEGFC (200ng)
J
APLN+VEGFC (40ng)
K
APLN+ VEGFC (20ng)
L
Ctrl limb (200ng)
200ng
40ng
20ng
VEGFC
APLN
APLN+VEGFC
M
limb CTL
limb LD
p=0.02
p=0.01
Limb diameter (mm)
D0 D7 D10 D14 D21
N
p=0.04
p=0.0007
ns
O
p=0.1
ns
P
p=0.1
p=0.1
ns

Figure EV5. VEGF-C-, APLN, and APLN-VEGF-C Lf dose response.

(**A**) Quantification of proximal limb swelling at 7 and 14 days after surgery on control limb, LD limb or LD treated with TSSM. Data represent mean ± SEM ($n = 5$) (**$P < 0.001$, two-way ANOVA). (**B**) Representatives images of lymphangiography from mice treated with TSSM. Scale bar: 1 mm. (**C–E**) Quantification of proximal limb swelling 7 and 14 days after surgery on control or LD treated with 200 ng (**C**), 40 ng (**D**), or 20 ng (**E**) of VEGF-C LentiFlash® vector. Data represent mean ± SEM ($n = 5$) (*$P < 0.05$, *$P < 0.001$, two-way ANOVA). (**F–H**) Quantification of proximal limb swelling 7 and 14 days after surgery on control or LD treated with 200 ng (**F**), 40 ng (**G**), or 20 ng (**H**) of APLN LentiFlash® vector. Data represent mean ± SEM ($n = 5$) (*$P < 0.05$, *$P < 0.001$, two-way ANOVA). (**I–K**). Quantification of proximal limb swelling 7 and 14 days after surgery on control or LD treated with 200 ng (**I**), 40 ng (**J**), or 20 ng (**K**) of APLN-VEGF-C LentiFlash® vector. Data represent mean ± SEM ($n = 5$) (*$P < 0.05$, *$P < 0.001$, two-way ANOVA). (**L**) Representatives images of lymphangiography from mice treated with 200 ng, 40 ng, or 20 ng VEGF-C-, APLN-, or APLN-VEGF-C LentiFlash® vectors. Scale bar: 1 mm. (**M**) Quantification of proximal limb swelling in mice treated with TSSM. Data represent mean ± SEM ($n = 5$) (**$P < 0.001$, two-way ANOVA). (**N**) Quantification of proximal limb swelling in mice treated with APLN-VEGF-C LentiFlash® vector Batch 1 after LD development (10 days post surgery). Data represent mean ± SEM ($n = 5$) (two-way ANOVA). (**O**) Quantification of proximal limb swelling in mice treated with APLN-VEGF-C LentiFlash® vector Batch 2 after LD development (10 days post surgery). Data represent mean ± SEM ($n = 5$) (two-way ANOVA). (**P**) Quantification of proximal limb swelling in mice treated with APLN-VEGF-C LentiFlash® vector Batch 3 after LD development (10 days post surgery). Data represent mean ± SEM ($n = 5$) (two-way ANOVA).

© The Author(s)
